# Colors for Resources: Reward-Linked Visual Displays in Orchids

**DOI:** 10.3390/plants15010154

**Published:** 2026-01-04

**Authors:** Gabriel Coimbra, Carlos E. Pereira Nunes, Pedro J. Bergamo, João M. R. B. V. Aguiar, Leandro Freitas

**Affiliations:** 1 Jardim Botânico do Rio de Janeiro, Rio de Janeiro 22460-030, RJ, Brazil; gabrielrocha@jbrj.gov.br; 2 Kunming Institute of Botany, Chinese Academy of Sciences, Kunming 650204, China; 3 Departamento de Biodiversidade, Universidade Estadual Paulista, Rio Claro 13506-900, SP, Brazil; 4 Faculdade de Filosofia, Ciência e Letras de Ribeirão Preto, Universidade de São Paulo, Ribeirão Preto 14040-900, SP, Brazil

**Keywords:** plant-animal communication, bee pollination, flower color, flower size, reward strategy, intrafloral modularity, flower integration, bee intertegular span

## Abstract

Pollination syndromes reflect the convergence of floral traits among plants sharing the same pollinator guild. However, bee-pollinated orchids exhibit striking variation in color and size. This diversity reflects the multiple reward strategies that evolved within the family, each interacting differently with bee sensory biases. Here, we tested whether the complex floral visual displays of orchids differ in signal identity and intensity among reward systems. We also considered intrafloral modularity, measured as the color differentiation among flower parts, and color–size integration. For this, we measured and modeled floral morphometric and reflectance data from sepals, petals, lip tips, and lip bases under bee vision from 95 tropical Epidendroid species to compare chromatic and achromatic contrasts, spectral purity, and mean reflectance across wavebands, plus flower and display size, among reward systems. Reward types included 19 food-deceptive, 8 nectar-offering, 10 oil-offering, 11 fragrance-offering, and 47 orchid species of unknown reward strategy. Principal component analyses on 34 color and 9 size variables summarized major gradients of visual trait variation: first component (19.1%) represented overall green-red reflectance and achromatic contrasts, whereas the second (16.5%) captured chromatic contrast–size covariation. Reward systems differed mostly in signal identity rather than signal intensity. Flower chromatic contrasts presented strong integration with flower size, while achromatic contrasts were negatively associated with display size. While deceptive and nectar-offering orchids tend toward larger solitary flowers with bluer and spectrally purer displays, oil- and fragrance-offering orchids tend toward smaller, brownish, or yellow to green flowers, with larger inflorescences. Rewardless orchids presented more achromatically conspicuous signals than rewarding orchids, but smaller displays. Orchid species clustered by reward both in PCA spaces and in bee hexagon color space. Deceptive orchids were typically associated with UV + White colors, oil orchids with UV + Yellow lip tips, and fragrance orchids with UV-Black lip bases and UV-Green lip tips. Together, these results indicate that orchid reward systems promote qualitative rather than quantitative differentiation in visual signals, integrating display color and size. These long-evolved distinct signals potentially enable foraging bees to discriminate among resource types within the community floral market. Our results demonstrate that color and flower display size are important predictors of reward strategy, likely used by foraging bees for phenotype-reward associations, thus mediating the evolution of floral signals.

## 1. Introduction

Most flowering plants depend on animals for pollen transfer [1], and bees are among the most frequent pollinators in both temperate and tropical ecosystems [2]. In orchids, bees pollinate half of all species studied so far [3]. To attract animal vectors and ensure effective pollination, plants rely on multiple flower signals such as scent [4], size [5], shape [6], and color [7], which are often modulated by the sensory capabilities and preferences of their pollinators [8,9]. For this, such traits are commonly interpreted within the framework of pollination syndromes [10], which predict convergence of floral signals among plants pollinated by the same guild of animals [8,10], as demonstrated in broad-scale studies [7,11,12,13]. For example, bee-pollinated flowers generally present colors with reflectance inflection points at regions of the spectrum optimal for bee vision [7,11,12,13]. However, in one of the greatest angiosperm families, the Orchidaceae, bee-pollinated species present remarkable diversity in flower form and display, challenging the expectation of strict convergence [14].

One explanation for this diversity is the wide range of reward systems in orchids [3,6,15]. While many angiosperms provide nectar or pollen as their primary resource [16], orchids have diversified into multiple strategies [3]. Some species produce nectar, a widespread and generalist reward employed by 51% of rewarding species [3,16]. The other 15% of rewarding orchids [3], such as Oncidiinae, offer floral oils [6,14,17], which are collected mainly by female Centridini or Tapinotaspidini bees and used in nest construction and larval provisioning [14,18]. Catasetinae provide floral perfumes [4,19,20], accounting for 24% of rewarding orchids [3], which are collected by male Euglossini bees that gather volatile compounds for courtship behavior [21]. These systems coexist within the family and illustrate the evolutionary flexibility of orchids in shaping pollinator interactions, evolving in different contexts of receiver bias [8,22], exploring innate preferences that outdate the evolution of flower color [8]. For this, just as flowers pollinated by different pollinator guilds converge into pollination syndromes, flowers pollinated by the same guild but presenting different types of rewards might be sharing the same signals as well [4,6]. For example, oil flowers from Oncidiinae have been shown to converge with much more rewarding Malpighiaceae, presenting color and shape more similar to them than expected by chance [6]. The visual signals of other rewarding orchids remain unexplored, such as those offering perfumes, whose olfactory signals are largely studied [4,22,23].

Since bees use different kinds of flower and non-flower resources [24,25], the same bee species might pollinate nectar-offering, oil-offering, and deceptive flowers throughout their lifetime. At the same time, plants would benefit from displaying shared signals with other floral or non-floral resources, facilitating reward-signal learning by bees with specific foraging schedules [6,25,26], promoting intraspecific behavioral niche partitioning and allowing enhanced pollinator sharing [27]. These systems evolved in different environmental and evolutionary contexts, nectar being the most ancient and well-established, while oil and fragrance-offering systems date back to approximately 70 million years [14]. Each one of these systems may have evolved as sensory traps, taking advantage of the sensory bias of bees, i.e., their innate or conditional color and size preferences [28]. In other words, these flowers exhibit color signals that had already evolved in response to other stimuli, such as in other rewarding species or non-floral material resources. For example, fragrance-offering orchids present cavity-looking lips, dark spots and yellow patterns that have been hypothesized to mimic Euglossini nests, where males use to offer fragrances for courtship behavior [22,29]. Such variation indicates that reward strategy may be a critical axis of orchid diversification, potentially influencing the evolution of floral traits such as color and size signals.

In addition to reward type, orchids also vary in the presence of reward, since 46% of them are deceptive, offering none [3]. The most studied rewardless system is sexual deceit, overrepresented by Orchidoideae orchids from temperate and subtropical regions [30,31], except for the understudied giant *Lepanthes* in the tropics [32]. In sexual deceit, flowers mimic receptive females and are pollinated by naïve males in an attempt to copulate [31,33]. Sexually deceptive orchids both visually and olfactorily mimic the female of their model Hymenopteran species by resembling them in shape, color, and scent, through extremely species-specific pseudo-pheromones [31,33]. In these systems, mimicking signals are often exaggerated, in both size and scent emission magnitude, matching the preferences of naïve pollinators [34,35]. The other most common rewardless system in orchids, accounting for 28% of all species, is food deception, which is much more prevalent in the tropics [3,35]. Food-deceptive orchids are pollinated by attracting naïve insects with signals that resemble rewarding flowers but without offering any food resources [15,36,37]. While sexual deceit is primarily scent-based, food deception is generally visually based, lacking olfactory cues [15,35], which might be easier to learn to avoid. Despite the importance of visual signals for food deception, there are no studies on a large scale, being generally limited to study cases of putative mimicry [38,39], also shown to exaggerate their model signals [38]. Thus, it remains unknown whether orchids lacking specific models display general food signals or exaggerate them in color and size.

Another important driver of visual trait evolution is how different aspects of the floral display are coordinated. Orchids frequently combine multiple color patches, or flower color modules, such as sepals, petals, and different regions of the lip petal [40], which may play distinct roles in attraction and guidance. Whether these modules function independently or as integrated units is an open question. Likewise, the relationship between floral size and other components of the display has rarely been addressed at a broad scale, despite size and intrafloral color variability being major determinants of pollinator attraction and detectability [41]. This might also differ among reward systems as rewards are presented in different forms. While nectar is often concealed in deep flowers, oils are offered in calli at the base or laterals of the lips in open flowers [17,42]. Fragrance-offering species also generally present open flowers (except for *Coryanthes* and pistillate flowers of *Catasetum*, which are cavity-looking), and osmophores not restricted to the lip bases [43]. Due to this structural diversity, flower modules may play different signaling roles in guiding bees in each system. Since bees are known to use achromatic cues at long distances and chromatic cues at short distances [41], we hypothesize that some flower modules, positioned more externally and more predominant in the display, are associated with long-distance attraction (sepals and petals), while inner modules aid in short-range guidance (lip tips and bases). Thus, we expected to find higher achromatic contrasts for sepals and petals, and higher chromatic contrasts for lip tips and bases, or higher chromatic intrafloral contrasts and higher achromatic extrafloral contrasts. In this context, the diversity of orchid reward systems and the flower structures associated with them offers an excellent opportunity to address these questions. If reward type influences how flowers advertise to pollinators, then we might expect differences in the degree of intrafloral modularity and in the strength of color–size integration across systems. Alternatively, modularity and integration could represent more general features of orchid floral biology, independent of reward.

**Figure 1:**
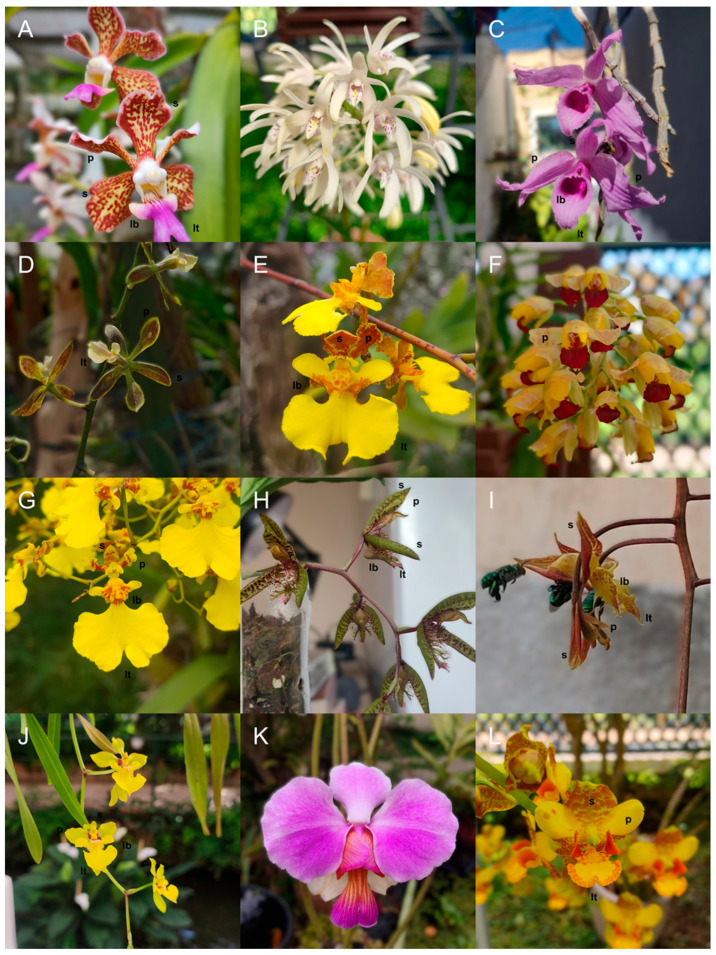
Orchid species sampled at the Rio de Janeiro Botanical Garden and G.C.’s personal collection, sorted by reward strategy: deceit, *Vanda tricolor* (**A**) and *Dendrobium speciosum* (**B**); nectar-, *Dendrobium anosmum* (**C**) and *Encyclia patens* (**D**); oil-, *Trichocentrum cepula* (**E**), *Gomesa echinata* (**F**) and *Gomesa flexuosa* (**G**); and fragrance-offering: *Catasetum multifidum* (**H**) and *Gongora bufonia* (**I**). Species with unknown reward systems: *Gomesa uniflora* (**J**), *Papilionanthe teres* (**K**) and *Cyrtopodium gigas* (**L**). Lower-case black letters indicate the flower color modules analyzed in this study: sepals (s), petals (p), lip tips (lt) and lip bases (lb).

Here, we analyzed 95 species of tropical bee-pollinated epidendroid orchids (Figure 1) to demonstrate how variability can be explained by different visual strategies under different reward systems, including the interplay of modularity and color–size integration. We evaluated differences in visual trait attributes, including color modeled to bee vision and morphometric data, among reward systems comparing nectar (*n* = 8 spp.), oil (*n* = 10), fragrance (*n* = 11), and food-deceptive (*n* = 19) orchids, including unknown reward systems (*n* = 47) as a null comparison. By integrating a large dataset of reflectance spectra and morphological measurements, we provide a large-scale assessment of how reward diversity influences color signals, modularity, and integration. Our findings shed light on the evolutionary interplay between reward systems and floral displays, offering new perspectives on how orchids achieve and maintain their extraordinary diversity.

## 2. Results

**Figure 2:**
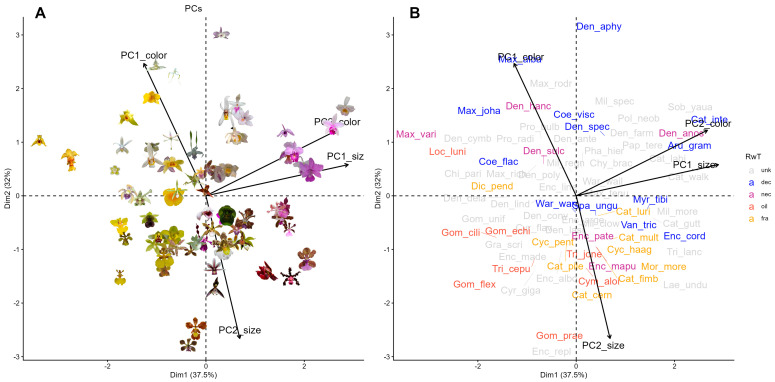
PCA biplot of four principal components from PCA_color_ and PCA_size_, showing the multivariate distribution of bee-pollinated orchid species and the contribution of each PC. *PC1_color_* describes achromatic contrasts, whereas *PC2_color_* describes chromatic contrasts and spectral purity. *PC1_size_* summarizes flower size, whereas *PC2_size_* summarizes inflorescence size. (**A**) Depicts the position of each orchid species with their respective pictures in PCA space, while abbreviated species names (refer to Table A1 for full names) are mapped into the same space in (**B**), colored by reward type (*RwT*): unknown (unk), deceptive (dec), nectar-offering (nec), oil-offering (oil) and fragrance-offering (fra).

Global PCA combining all 34 color and 9 size variables explained 35.1% of total variability in the first two components (*PC1_global_* = 19.1%, *PC2_global_* = 16.0%; Table S1, Table A1 and Table A2). *PC1_global_* was dominated by achromatic contrast and green-red reflectance of sepals and petals, while *PC2_global_* reflected covariation between spectral purity and display size (flower area, radius, and inflorescence length) and blue reflectance. PCA_color_ explained 38.7% of variability in the first two components, separating flowers by achromatic contrast (*PC1_color_* = 23.5%) vs. chromatic contrast and spectral purity (*PC2_color_* = 15.2%). PCA_size_ explained 75.1% of variability in the first two components (*PC1_size_* = 52.8%, *PC2_size_* = 22.3%), summarizing variation in flower and display size, respectively. Our final PCA, performed on the four PCs of the two previous analyses (PCA_color_ + PCA_size_), revealed strong color–size integration (Figure 2), with a positive association between *PC2_color_* (chromatic contrast) and *PC1_size_* (flower size) on the first axis (*PC1_final_* = 37.5%, Table 1) and a negative association between *PC2_size_* (display size) and *PC1_color_* (achromatic contrast) on the second axis (*PC2_final_* = 32%).

### 2.1. Signal Identity Among Reward Types

**Figure 3:**
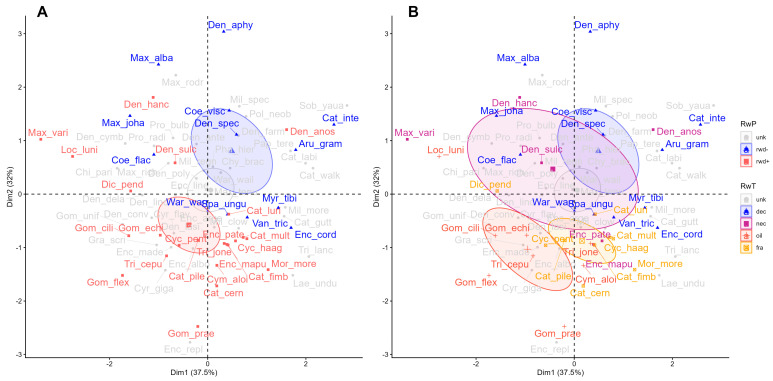
PCA biplot of all color and size PCs showing the distribution of bee-pollinated orchid species according to reward presence (**A**) and type (**B**) in PCA space. Colors indicate reward categories: deception (dec), nectar (nec), oil (oil), fragrance (fra), and unknown (unk). Each point represents one species projected in the multivariate space defined by the first two principal components. The first axis (*Dim1*, 19.1%) represents *PC1_final_*, dominated by chromatic contrast and flower size, while the second axis (*Dim2*, 16.0%) represents *PC2_final_* (achromatic contrast and display size). Ellipses represent 95% confidence intervals for each reward group. Species names are abbreviated for simplicity, refer to Table A1 for full names.

Multivariate analyses revealed differentiation in floral signals among reward systems across both PCA and bee color hexagon spaces. In PCA_final_, species clustered significantly both by reward presence (*F* = 4.45, *R*^2^ = 0.11, *p* = 0.005; Figure 3, Table 2) and reward type (*F* = 43.80, *R*^2^ = 0.18, *p* = 0.005). Rewardless orchids differed from rewarding species in general (*RwP*: *F* = 8.27, *R*^2^ = 0.19, *p* = 0.001; Table S2); specifically, from oil- (*F* = 10.75, *R*^2^ = 0.36, *p* = 0.001) and fragrance-offering species (*F* = 7.41, *R*^2^ = 0.27, *p* = 0.004), but not from nectariferous species (*F* = 1.21, *R^2^ =* 0.07, *p* = 0.31). PCA_color_ followed a similar pattern (*F* = 3.07–4.97, *R*^2^ = 0.12–0.15, *p* = 0.003–0.006). For PCA_size_, however, orchids differed only among reward types (*R*^2^ = 0.11, *F* = 2.79, *p* = 0.010) but not reward presence (*F* = 2.16, *R*^2^ = 0.04, *p* = 0.076). Specifically, oil-offering species differed from both nectar-offering (*F* = 5.75, *R^2^* = 0.26, *p* = 0.002) and deceptive species (*F* = 5.81, *R*^2^ = 0.18, *p* = 0.006).

**Figure 4:**
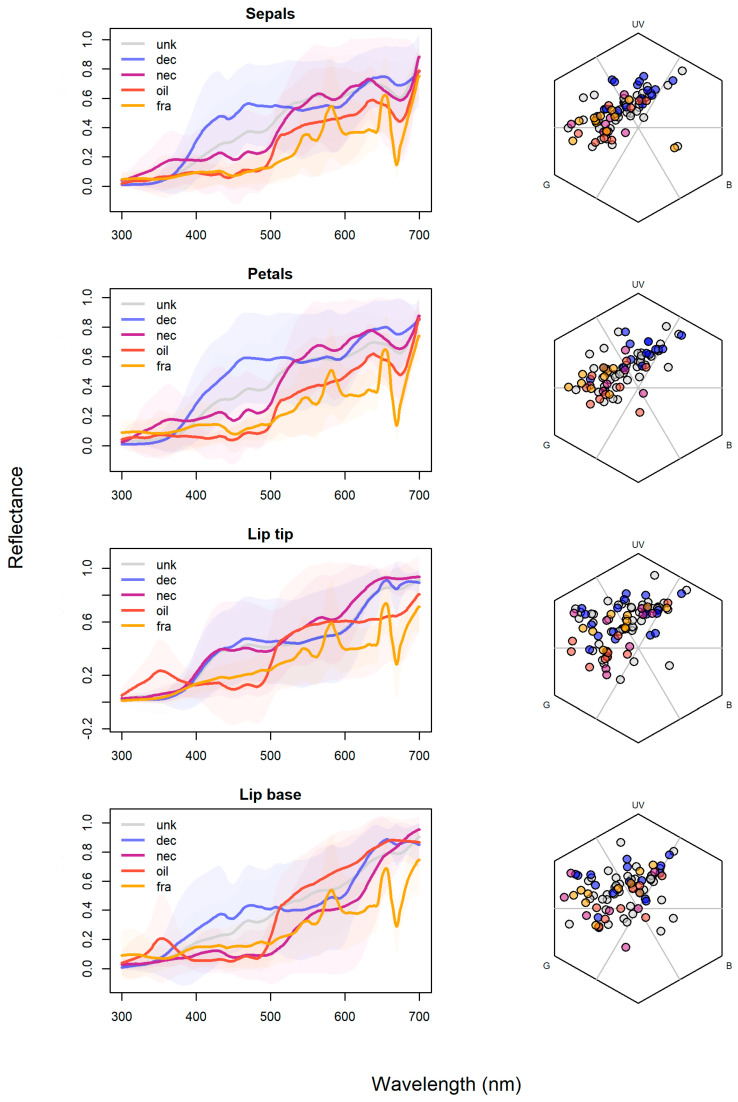
Mean reflectance spectra (left column) and positions in the bee hexagonal color space (right column) for floral color modules of tropical bee-pollinated orchids, grouped by reward type. Rows show, from top to bottom, sepals, petals, lip tips and lip bases. In the spectral plots, solid lines represent the mean reflectance of species within each reward system: deceptive (dec), nectar- (nec), oil- (oil), and fragrance-offering species (fra), plus species with unknown rewards (unk). Shaded areas indicate among-species variation. In the hexagonal diagrams, each point is a species, plotted according to its bee-perceived color coordinates for the corresponding floral module, with symbol colors matching reward systems and axes labeled by the three bee photoreceptor channels: ultraviolet (UV), blue (B) and green (G).

Analyses in bee hexagon color space also confirmed significant clustering by reward presence (*F* = 2.79–11.08, *R*^2^ = 0.06–0.23, *p* = 0.001–0.031; Figure 4) and reward type (*F* = 1.93–6.30, *R*^2^ = 0.09–0.25, *p* = 0.035–0.075), whereby lip bases were the only flower color module not to form clusters by reward type (*F* = 1.93, *R*^2^ = 0.09, *p* = 0.075). In sepals, rewardless species clustered next to the UV section of the hexagon, differing from rewarding species as a group (*RwP*: *F* = 17.65, *R*^2^ = 0.31, *p* = 0.001) and from nectar- (*F* = 9.25, *R*^2^ = 0.32, *p* = 0.005), oil- (*F* = 9.23, *R*^2^ = 0.29, *p* = 0.005) and fragrance-offering species (*F* = 12.02, *R*^2^ = 0.32, *p* = 0.001), which did not differ between them (*F* = 0.01–0.24, *R*^2^ = 0.001–0.017, *p* = 0.77–0.99), clustering around the Green-UV hexagon section. In petals, rewardless orchids differed from rewarding orchids (*RwP*: *F* = 22.86, *R*^2^ = 0.38, *p* = 0.001) and from nectar- (*F* = 6.20, *R*^2^ = 0.25, *p* = 0.017), oil- (*F* = 12.62, *R*^2^ = 0.36, *p* = 0.002) and fragrance-offering orchids (*F* = 20.94, *R*^2^ = 0.49, *p* = 0.001), which did not differ among them (*F* = 0.43–3.17, *R*^2^ = 0.03–0.20, *p* = 0.07–0.65), following the same UV-Green vs. UV section divergence as sepals. In lip tips, rewardless species also differed from rewarding species in the hexagon (*RwP*: *F* = 4.51, *R*^2^ = 0.09, *p* = 0.02), but only from oil-offering species (*F* = 7.84, *R*^2^ = 0.23, *p* = 0.01). Lip bases were the least informative flower module, with species clustering only by reward presence (*F* = 3.85, *R*^2^ = 0.09, *p* = 0.03).

PERMDISP analyses confirmed that the significant PERMANOVA results were not caused by unequal within-group dispersion (Table S3). Dispersion did not differ among groups in PCA spaces (PCA_final_, PCA_color_, and PCA_size_) or bee hexagon analyses (*F* = 0.01–2.13, *p* = 0.09–1.00). These results validate our PERMANOVA findings, showing that the clustering by reward system reflects genuine differences in multivariate trait centroids rather than heterogeneity in trait variance among groups.

Chi-squared tests also revealed significant associations between reward type (*RwT*) and color categories across all floral modules analyzed (Figure S1): sepals (χ^2^ = 76.97, *p* = 0.006), petals (χ^2^ = 64.37, *p* = 0.026), lip tips (χ^2^ = 96.30, *p* < 0.001), and lip bases (χ^2^ = 76.40, *p* = 0.007).

In sepals, oil-rewarding species were overrepresented among UV-absorbing black flowers (hitherto “UV-Black”; standard residuals = 2.27, *p* = 0.023). In petals, similar patterns emerged: oil (*SR* = 2.39, *p* = 0.02) and fragrance species (*SR* = 2.06, *p* = 0.04) were more frequent among UV-Black colors, whereas deceptive orchids were the most frequent among UV + White (SR = 3.10, *p* = 0.01) or UV-Red hues (*SR* = 2.18, *p* = 0.03). In lip tips, oil species were again associated with dark colors (UV-Black, *SR* = 2.10, *p* = 0.04) but most strongly with UV + Yellow (*SR* = 4.15, *p* < 0.01). Fragrance orchids showed a strong positive association with UV-Green (*SR* = 5.53, *p* < 0.001), while nectar-offering species often displayed yellow lip tips (UV-Yellow, *SR* = 3.22, *p* < 0.01). A total of 16 species presented UV-Yellow lip bases, broadly distributed among reward types except fragrance-offering species (*SR* = −1.68). This was the floral module with the weakest association with reward system, though some patterns persisted: UV-reflecting pink (UV + Pink) in deceptive orchids (*SR* = 3.10, *p* < 0.01) and UV + Black and UV-Cyan in fragrance-offering orchids (both *SR* = 3.85 and *p* < 0.01).

### 2.2. Signal Intensity Among Reward Systems

**Figure 5:**
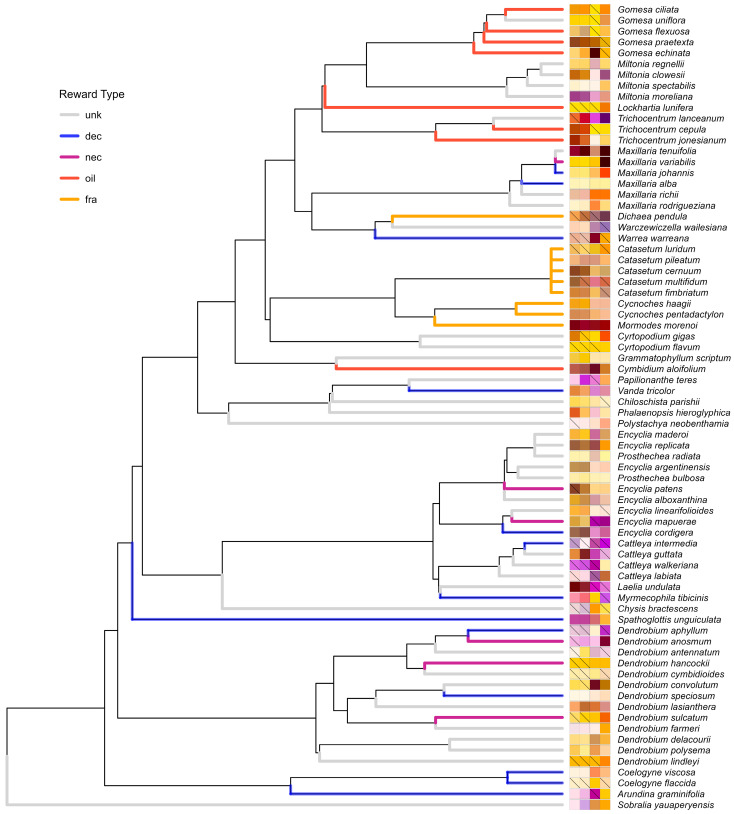
Phylogenetic distribution of intrafloral color patterns in bee-pollinated tropical orchids generated according to Jin and Qian (2022) [44]. For each species, the horizontal bar on the right depicts a square for each of the four flower color modules sampled, from left to right: sepals, petals, lip tip, and lip base. Colors are rendered as an RGB approximation in human vision from reflectance spectra (300–700 nm) measured from cultivated individuals from the Rio de Janeiro Botanical Garden and G.C.’s personal collection. Crossed squares indicate UV reflectance equal or above 10%. Terminal branches are colored according to the reward type of each species: unknown (unk), deceptive (dec), nectar- (nec), oil- (oil) or fragrance-offering (fra).

Phylogenetic analyses demonstrated weak constraints on the evolution of flower color and size signals, with great variability within closely related taxa (Figure 5). Blomberg’s *K* values were consistently close to zero (*K* = 0.001–0.55) and non-significant for all traits, indicating no phylogenetic signal across species (Table 3).

Phylogenetic ANOVAs indicated limited quantitative differences in visual signals (PC scores) among reward systems. The only significant difference was in *PC2_final_*, which summarizes the negative association between achromatic contrast and display size, among rewarding and rewardless species (*PC2_final_* by *RwP*, *p* = 0.03, *F* = 7.38; Figure S2B,H, Table S4), where rewardless species present flowers achromatically more conspicuous but smaller displays. All other comparisons in mean PC scores, whether considering color or size separately, were non-significant (*F* = 0.99–6.28, *p* = 0.10–0.60), indicating no further detectable differences in visual signal intensity among reward systems after accounting for shared evolutionary history.

### 2.3. Flower Integration and Modularity

**Figure 6:**
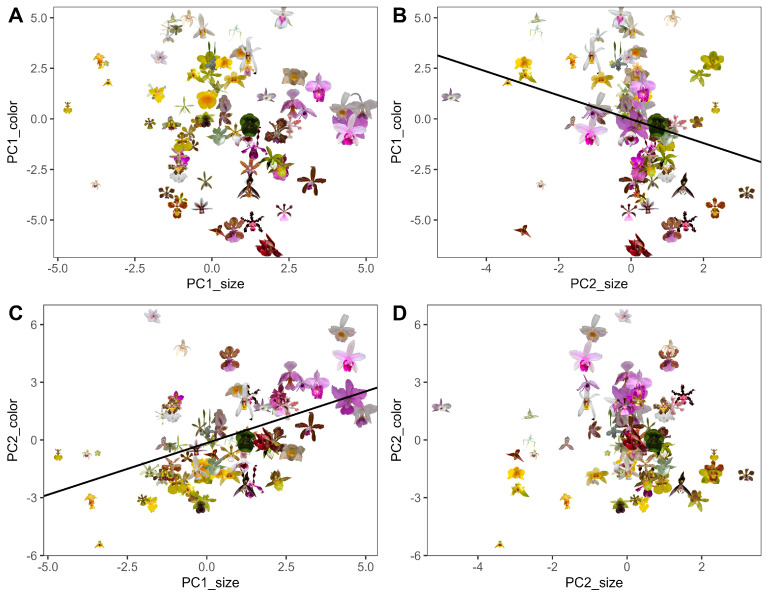
Associations between color and size principal components in bee-pollinated orchids. Each point represents a species positioned by its standardized scores on color (*y*-axis) and size (*x*-axis) principal components, with images illustrating floral diversity (scaled). Panels show pairwise correlations between color and size dimensions: (**A**) *PC1_color_* (achromatic contrast) vs. *PC1_size_* (flower size), (**B**) PC1_color_ vs. PC2_size_ (display size), (**C**) PC2_color_ (chromatic contrast) vs. PC1_size_, and (**D**) PC2_color_ vs. PC2_size_.

Linear regressions confirmed significant integration between flower color and size dimensions. *PC2_color_*, representing chromatic contrast, was positively associated with *PC1_size_*, which represents flower size (*R*^2^ = 0.22; *F* = 20.12; *p* < 0.001; Figure 6), whereas *PC1_color_* (achromatic contrast) showed a negative association with *PC2_size_*, representing display size (*R*^2^ = 0.086, *F* = 6.76, *p* = 0.011). No other color–size models were significant (Table 4).

Mean pollinator body size (*ITS*, Tables S6 and S7) was not associated with any size PCs (*F* = 3.49–3.65, *R*^2^ = 0.10, *p* = 0.06–0.07; Figure S3), but rather negatively associated with both *PC2_final_* (achromatic contrast vs. display size; *F* = 5.14, *R*^2^ = 0.18, *p* = 0.03) and *PC1_color_* (achromatic contrast; *F* = 9.37, *R*^2^ = 0.29, *p* = 0.01). These results indicate that species with higher achromatic contrasts and smaller displays are associated with larger bee species, although *ITS* did not differ between reward presence (*F* = 0.29, *p* = 0.74) or among reward types (*F* = 0.65, *p* = 0.62; Figure S4).

**Figure 7:**
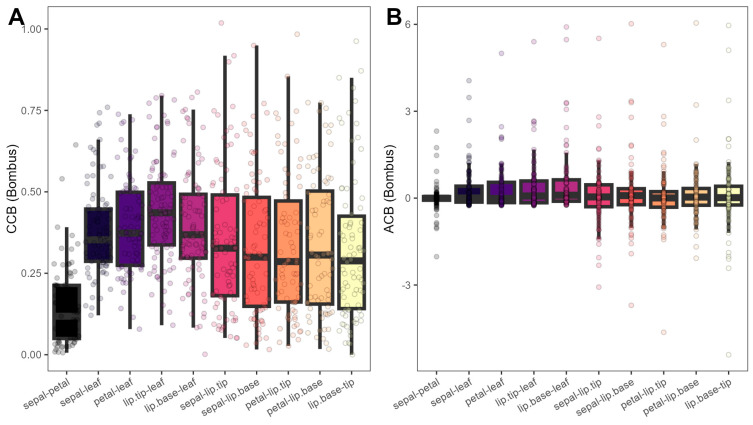
Bee chromatic (**A**) and achromatic contrasts (**B**) differences between different pairs of floral color modules.

Linear mixed-effects models indicated that chromatic and achromatic contrasts between floral modules were associated both with the identity of the flower module pair (Figure 7, Table S5), as well as with floral and display sizes (*F* = 1.64–13.85, *p* = 0.00–0.20; Figure S5), with no effect of reward presence or type (*F* = 0.93–1.57, *p* = 0.21–0.45), confirming intrafloral modularity and integration regardless of reward system. *PC1_size_* had a positive effect on chromatic contrasts (*F* = 12.70–12.94, *p* < 0.0001; Figure S5A), whereas *PC2_size_* was negatively associated with achromatic contrasts (*F* = 9.01–13.85, *p* < 0.001; Figure S5B). Pair identity was significant both on chromatic (*F* = 18.69–18.71, *p* < 0.0001; Figure 7A), where sepal-petal contrasts were lower than all others (*t* = −11.63–−6.81, *p* < 0.0001), and achromatic contrasts (*F* = 2.36–2.38, *p* = 0.011–0.012; Figure 7B), where only the lip base-leaf pair displayed higher contrasts than the sepal-petal pair (*t* = −3.35, *p* = 0.027).

## 3. Discussion

Here, we asked whether reward type modulates the evolution of visual signals in Orchidaceae, demonstrating how flower color and size are integrated and consistently differ among reward systems when analyzed jointly, both in signal intensity and signal identity. Conversely, intrafloral modularity and integration appear to represent general organizational patterns decoupled from reward strategy, with coordinated integration among long-distance signaling traits and, separately, among short-range signaling traits. In this study, using cultivated individuals and literature data, we assembled the largest dataset combining spectral and morphological traits of bee-pollinated orchids, fully considering their complex displays by sampling four flower color modules per species. We address for the first time the visual signals of fragrance-offering orchids and test the long-standing hypothesis of signal exaggeration by rewardless orchids on a large scale.

Most of the studies on the visual ecology of reward-phenotype correlations have focused on signal reliability, which is generally considered the informative potential of signal intensity on the quantity of reward offered by the plant [45], be it nectar or pollen [46,47,48]. We present here an innovative approach, framed as a qualitative assessment of signal reliability, finding that rewarding orchids honestly signal their reward types through specific visual signals while rewardless orchids exploit that association, presenting generalized rewarding signals partially convergent with nectar-rewarding species. We find strong evidence for signal identity differentiation among reward systems, and mixed evidence for signal intensity exaggeration by rewardless species.

Considering signal identity, multiple studies reported clustering of color loci of bee-pollinated plants in the Blue-Green section of the color hexagon [11,12,13,49,50,51]. However, all were community-wise studies with sympatric species, considering one color per display, which helps explain why the color loci of orchids in our sample mostly cluster in the UV-Green section, regardless of color module (Figure 4). Papadopulos et al. (2013) [6] found that orchids, and especially oil-offering Oncidiinae and their putative Malpighiaceae model species, clustered in the UV-Green section of the hexagon as well, similar to oil orchids in our sample. Thus, our results extend previous evidence of a reward-linked oil syndrome, with large displays but small, achromatically inconspicuous flowers that differ from food-deceptive and nectar-offering species, partially overlapping with fragrance-offering orchids. Deceptive species, in turn, clustered around the UV section, another section rarely occupied by other angiosperm flowers (Papadopulos et al., 2013) [6], indicating that some rewardless orchids may exploit signals that are novel, and possibly more appealing to naïve bees in their communities [34]. This is corroborated phylogenetically as shown in Figure 5 where food-deceptive/nectar-offering genera such as *Cattleya* and *Dendrobium* present more incidence of UV in multiple flower modules, and bluer hues, while oil- and fragrance-offering genera, such as *Gomesa* and *Catasetum*, usually have UV restricted to a single flower module, besides redder hues.

Considering signal intensity, such as color and size traits, none presented phylogenetic constraints, meaning they are more related to ecological factors than to their shared evolutionary history. This confirms that visual traits are extremely evolutionarily labile in plants [52]. However, visual trait evolution seems to be coordinated, or traded off, between flower display color and size. Since phenotypic integration is usually studied at the individual level, this has only been demonstrated in multispecies tests considering the predominant color against the background and flower depth [52,53,54]. In all three cases, deeper, larger flowers were less conspicuous in bee vision, both in the achromatic and chromatic channels. In our sample, we found paradoxical effects depending on the visual channel considered. While chromatic contrast scaled up along with flower size, achromatic contrast did not. When display size scaled up, however, achromatic contrasts decreased, while chromatic contrasts did not change. We believe this to be evidence of display partitioning, whereby two sets of traits commonly attributed to similar functions are integrated, while being decoupled from other unrelated traits. The achromatic channel, long considered important for long-distance detection, is integrated with display size with a negative association, probably representing a trade-off, where achromatic contrasts become less important if displays are large enough to signal from a distance. At short range, however, larger flowers require extra guidance for pollinators to correctly find resources and elicit correct positioning for pollination, so chromatic contrasts, important at short distances, must scale up accordingly. Several species combined UV-absorbing white sepals and petals with darker, UV-absorbing red or yellow lip bases, respectively the most and least conspicuous color categories in bee vision [52,55]). Consequently, display-background contrasts were consistently higher than intrafloral contrasts (Figure S5), hinting at stronger selection for detectability against the leaf background. This makes sense because long-distance visual detection is, after scent, crucial for plant resource location by bees [41,56]. This long-distance signaling role of more external reproductive parts as opposed to more internal reproductive parts has also been shown in other bee- and hummingbird-pollinated lineages [54,57]. Thus, we confirm our hypothesis that tepals function primarily as long-distance signals, subject to stronger selection for conspicuousness against the foliage. Lip bases and tips, on the other hand, may have been selected as short-range guides, reward mimics, or protection to reproductive structures from radiation [40,58,59]. This suggests that specific combinations of signals may be required to guide bees for distinct tasks, such as collecting oil in the lip base or laterals of oil-offering orchids [60] or positioning male Euglossini bees to better gather floral fragrances [61]. These signal combinations are subject to unique arrays of the selection factors just mentioned for each species, generating unique signal combinations in each species. This uniqueness might play a role in speciation and reproductive isolation by becoming selectively attractive to pollinators during pollinator shifts. This might explain why floral integration and modularity did not differ among reward systems, despite their seemingly contrasting flower display structures.

In Mediterranean nectar-offering species, Kantsa et al. (2017) [51] found that nectar-offering species were spectrally purer than non-nectariferous species. Although in that study, non-nectariferous species were not necessarily food-deceptive like the orchids we analyzed here, they presented inconspicuous colors such as UV-Red and relied mostly on olfactory cues, more like the fragrance-offering species in our sample. Nectar-offering orchids showed intermediate visual signals between deceptive and specialized rewarding systems (oil and fragrance). They often combined UV-White tepals with more saturated or yellowish lip regions that act as localized guides, which are more generalized signals that may be attractive for a wide array of food-foraging bees [62]. Their multivariate positions overlapped partly with deceptive orchids in the color–size space, further supporting the idea that deceptive species exploit the perceptual space occupied by nectar flowers. Even though deceptive species produce prominent UV reflection, they exhibited hues similar to the general visual spectrum of rewarding communities, especially white and yellow colors. This is consistent with food deception targeting naïve or exploratory bees that respond to generalized rewarding cues of nectar flowers, and with deceptive orchids relying on perceptual resemblance to co-flowering models rather than on signal amplification [63]. Only in the achromatic channel did rewardless orchids present higher signal intensity than rewarding ones, but such intensity is traded off with smaller displays. Low population densities in tropical orchids, as shown by their strong pollen limitation, reduce their potential as reliable food sources [15]. Under these conditions, generalized, conspicuous colors can function as opportunities within a reward landscape, and occasional successful deceptive visits may suffice to ensure reproduction [36]).

Oil flowers, particularly in Oncidiinae, with their brownish, UV-Black tepals and UV + Yellow lips in dark UV-Black or UV + Yellow, converge to the Malpighiaceae oil-flower syndrome [64,65]. Moreover, oil flowers offer oils frequently experience illegitimate visitation by non-pollinating oil thieves [60] that likely select for reduced visual conspicuousness to non-target visitors [52]. In parallel, fragrance-offering orchids seem to rely on volatile compounds that act as both the attractant and the reward, often presenting inconspicuous color patterns. This perhaps indicates undirected visual signaling toward reward location and primary reliance on olfactory cues, as osmophores are spread across tepals and other floral structures [43]. Since fragrance rewards are linked to courtship rather than feeding, visual displays may have a lower importance in comparison with olfactory cues. The visual signals of fragrance-offering species suggest that selection acts more strongly on olfactory cues and reward-offering location than on visual displays, which rather resemble decaying wood or tree barks: ancient sources of volatiles also used by these bees [65].

Some limitations in our study might have limited our understanding of visual signal evolution in orchids. Taking measures of bases and tips of all floral structures, not just lips, would have better demonstrated intrafloral modularity, as did Aguiar et al. (2020) [40]. Studies integrating flower shape [6] and flower scent with pollinator behavior might further advance the hypothesis of reward syndromes. Such approaches will improve our understanding of qualitative signal reliability in bee-pollinated plants and how it is modulated by bee foraging behavior.

## 4. Materials and Methods

All the following analyses were conducted in R version 4.5.2 [66] using RStudio (version 2025.09.2+418; [67]) and the latest available versions of the following packages: vegan [68], phytools [69], pavo [70], lme4 [71], V.PhyloMaker2 [44] and FactoMineR [72].

### 4.1. Databases

To investigate the visual signals of bee-pollinated tropical orchid species, which present populations too sparse or inaccessible to sample on a large scale, we sampled cultivated individuals from the Orchidarium of the Rio de Janeiro Botanical Garden (JBRJ, *n* = 83 species) and from G.C.’s personal collection (*n* = 18). We considered all species with at least three flowers available on monthly visits from August 2017 to November 2018, April 2022 to July 2023, and March 2024 to August 2025. Additionally, we included 8 species from C.E.P.N.’s personal database and 24 species from the Floral Reflectance Database [73]. We extracted data on flower visitors and floral rewards for each sampled species from Ackerman et al. (2023) [3]. From the total 133 orchid species sampled, 118 species were from tropical regions. We found data on floral rewards for 82 species, and on pollinator data for 61 species. From the total 133, we selected 95 tropical species, for which reward and pollinator data were available (48), plus 47 species with unknown reward systems, but from genera or subtribes dominated by Hymenopteran pollination [3] and thus also putatively bee-pollinated (see Table A1), such as *Bifrenaria* (2 out of 3 studied species in the genus are pollinated by bees), *Cattleya* (16/18), *Coelogyne* (10/11), *Cyrtopodium* (5/5), *Dendrobium* (19/24), *Encyclia* (5/5), *Gomesa* (9/9), *Laelia* (9/10), *Maxillaria* (7/7), *Phalaenopsis* (3/3), *Polystachya* (4/4), *Prosthechea* (3/3), *Sobralia* (5/6), *Trichocentrum* (12/12), and *Warczewiczella* (2/2).

Our final dataset contained 95 tropical putatively bee-pollinated Epidendroid orchid species (Figure 1, Table A1): 47 with unknown reward types, 19 food-deceptive, 8 nectar-offering, 10 oil-offering, and 11 fragrance-offering species. We, thus, used the category “unknown” as the reference level against which known reward systems (deceit, nectar, oil, and fragrance) were compared. We included 2 species with confirmed food trichomes (*Maxillaria crassifolia* and *Polystachya pubescens*) in the category “unknown” since they were too few to stand in a separate group. Considering geographic origin, we sampled 65 species from the Neotropics, 17 from Asia, 11 from Oceania, and two from Africa, comprising 7 tribes and 15 subtribes of the Epidendroideae subfamily (Figure 5).

We partitioned our dataset into three subsets: (i) a total of 95 species with a varying number of measured flower modules (at least one flower module, all morphometric measurements), used for size PCA and intrafloral modularity analysis; (ii) 74 species with complete observations (all four flower modules plus all morphometric measurements), used for global and color PCA; and (iii) 35 species with available data on pollinators, used for testing color–size integration with pollinator size.

### 4.2. Flower Size Signals

We measured petal, sepal, lip, and inflorescence lengths plus flower depth in mm with a digital caliper. We also took pictures (front and side shots) of three to six flowers per species against scaled white paper and then used the imageJ software (version 1.54g; [74]) to estimate the mean flower surface area in mm^2^. We also counted the number of flowers per inflorescence and the number of inflorescences per individual for each species (one individual per species).

To account for pollinator size in our analysis of flower size signals, we also estimated the mean body size of visitors for all species with available information in the literature. We used intertegular distances (i.e., the span between the posterior wing bases; hereafter ITS) extracted from the literature for each bee species as a proxy for bee body size [75]. Wherever ITS was not available for a given species, or visitors were not identified down to species level, we used the mean ITS for the genus, calculated from a compiled database of 744 bee species (Table S7).

### 4.3. Flower Color Signals

We took reflectance measurements at an angle of 45° using a portable spectrometer (USB 4000; Ocean Optics), barium sulfate (BaSO_4_) as a white standard and a black chamber as a black standard [76]. We took at least three reflectance measurements from each flower module: sepals, petals, lip tip, and lip base. To model the reflectance data of all flower color modules according to bee vision, we used the spectral sensitivities of *Bombus terrestris* L. (328, 428, and 536 nm; [77]), for it is a well-studied visual system closely related to tropical bumblebee species. We are confident this approximation is consistent with overall selective pressures from bees since Hymenopteran visual systems are usually well-conserved phylogenetically [77].

Using the peak sensitivities of *B. terrestris* in Chittka’s color hexagon [78], we applied the *pavo::vismodel* and *pavo::coldist* functions [70] using average leaf reflectances as the standard leaf background from Coimbra et al. (2020) [52] and the “forest shade” irradiance function as our standard illuminant, as most of our species are epiphytic flowering under tree canopies. This way, we were able to compute chromatic and achromatic contrasts against the background according to the visual systems of our model species *Bombus terrestris* [77]. We defined the chromatic contrast as the color distance between flower modules and the standard leaf background (*dS* from *pavo::coldist*), and the achromatic contrasts as the “green contrast”, computed using the green photoreceptor. We used luminance (*lum*) from *pavo::vismodel* in extrafloral contrasts and *dL* from *pavo::coldist* for intrafloral contrasts. We thus computed chromatic and achromatic contrasts among flower modules and leaves (all 10 combinations: leaf–sepal, leaf–petal, leaf–lip apex, leaf–lip base, lip apex–lip base, sepal–lip apex, sepal–lip base, petal–lip apex, petal–lip base, sepal-petal) for each species. We coded these combinations as a categorical variable *pair*. Both metrics are important because bees were shown to alternately use chromatic and achromatic cues when foraging, depending on visual angle [41]. We further computed spectral purity, a relevant metric in bee attraction by which some bees show innate biases for higher values [79]. Spectral purity for *B. terrestris* was quantified in the hexagon color space by first generating the monochromatic locus (hexagon boundary) from simulated monochromatic stimuli spanning 300–700 nm, which were projected using *pavo::vismodel* and *pavo::colspace* (“*hexagon*”). For each flower module spectrum measured, we calculated its distance to the achromatic center and identified the closest point on the monochromatic boundary. Spectral purity was then computed as the ratio between the module’s radial distance from the center and the distance from the center to the corresponding boundary point, yielding values near zero for weakly saturated colors and near one for highly saturated colors; all distances were Euclidean and computed in the *xy* space.

### 4.4. Variation in Flower Color and Size Signals

To summarize the main gradients of variation in floral visual traits, we conducted four separate principal component analyses (PCA) on standardized (z-scored) variables: (i) a global PCA, with all color and size variables (PCA_global_); (ii) a color PCA with 34 color variables (PCA_color_); (iii) a size PCA with nine floral display size variables (PCA_size_), and then a final PCA (PCA_final_) using the first two PCs of the two previous PCAs (*PC1_color_*, *PC2_color_*, *PC1_size_* and *PC2_size_*). We did this because of the uneven number of color and size variables, so color would not be given greater weight over size for variable number alone. Since we were unable to normalize intrafloral achromatic contrasts (*dL* from *pavo::coldist*), we included only extrafloral (background-display) achromatic contrasts (*lum* from *pavo::vismodel*) in our PCA analyses. We conducted analyses with *FactoMineR::PCA* retaining the first two components (PC1 and PC2) of each analysis. The six latter component scores were then merged into the species dataset and used as proxies of visual signal intensity in the remaining analyses.

### 4.5. Flower Signal Identity Among Reward Types

We classified the spectra of each color module sampled into discrete spectral categories following the methods in Coimbra et al. (2020) [52]. For this, we averaged the reflectance intensity of each flower color module across four bands of the spectrum: ultraviolet (from 301 to 400 nm), blue (401–500 nm), green (501–600 nm), and red (601–700 nm) and then assigned either “absorbing” (−) or “reflecting” (+) for each band according to thresholds selected based on the distribution of the data: 10% for the UV band, 30% for blue, 40% for green, and 60% for red. We also classified flowers reflecting in the green band with a difference ≥ 50% compared to the blue/red bands as green absorbing. Then, to examine categorical color–reward associations, we used Pearson chi-square tests with simulated *p*-values (B = 1 × 10^6^) for each floral module separately. Spectra were first grouped into discrete color classes (e.g., UV + White for UV-absorbing white, or UV + White for UV-reflecting white) based on bandwise reflectance thresholds. Contingency tables were built between reward type (*RwT*) and color category for sepals, petals, lip tips, and lip bases. For each test, we recorded global χ^2^ and *p*-values, as well as standardized residuals (*SR*) for each cell. Residuals quantified deviations from random expectations, identifying reward–color combinations occurring more (positive residuals) or less (negative residuals) frequently than expected by chance.

To evaluate multivariate differentiation and clustering in floral visual signals according to reward type, both in PCA and color hexagon coordinates, we used PERMANOVAs (*vegan::adonis2*) based on Euclidean distances (999 permutations). For PCA spaces, we fit three models at the species level: a final model including both color and size axes (PC1_final_, PC2_final_), a color model (PC1_color_, PC2_color_), and a size model (PC1_size_, PC2_size_), each using either reward presence (*RwP*) or reward type (*RwT*) as a grouping factor. We extracted *F*, *R*^2^, and *p*-values to assess clustering by reward system, followed by pairwise PERMANOVAs between reward groups. To ensure these differences were not driven by within-group heterogeneity, we tested multivariate homogeneity of dispersion using PERMDISP (*vegan::betadisper* and *vegan::permutest*) and post-hoc Tukey comparisons. The same framework was applied to the coordinates (x, y) of each flower module (sepals, petals, lip apex, and lip base) in the bee hexagon color space. These tests evaluated whether species with different reward types occupied distinct regions in the bee perceptual color space for each floral color module.

### 4.6. Flower Signal Intensity Among Reward Types

First, to account for shared ancestry, a phylogenetic tree of all sampled species was assembled using *V.PhyloMaker2::phylo.maker* under Scenario 2 (the one which performed best for our species, generating fewer polytomies) with the GBOTB.extended backbone. We computed phylogenetic signal as Blomberg’s K for each numeric trait with *phytools::phylosig* to test whether visual and morphological traits showed evolutionary conservatism.

We, then, used *phytools::phylANOVA* (5000 simulations) to test whether mean PCA scores differed among reward systems while controlling for shared ancestry. We considered both reward presence (*RwP*: rewardless, rewarding, and unknown) and reward type (*RwT*: deceit, nectar, oil, fragrance, and unknown).

### 4.7. Testing Color–Size Integration

Floral integration was analyzed using linear models (LMs) to test how color and size axes covary with each other and with pollinator body size (mean intertegular span, *ITS*). Ordinary least squares regressions were run for all color–size combinations and for color–size against mean ITS. Specifically, *PC1_color_* and *PC2_color_* were modeled as response variables against *PC1_size_* and *PC2_size_* as predictors to test color–size integration. Additional models tested the relationship between each PCA axis and *ITS* to evaluate whether larger flowers are associated with larger pollinators. For each model, we extracted slope estimates, standard errors, *t* statistics, *p*-values, and R^2^, verifying assumptions of residual normality and homoscedasticity.

### 4.8. Testing Intrafloral Modularity

Finally, intrafloral modularity was analyzed with linear mixed-effects models (LMMs) to determine how chromatic and achromatic contrasts vary among floral structures and whether these contrasts depend on size or reward systems. We used LMMs to account for the nestedness structure of our data, using more than one pair of modules per species in the same models. For this, each pair was coded as a categorical factor (*pair*), and species identity (*sp*) was included as a random effect. We fit LMMs with *lme4::lmer* and formulas in the following format: *contrast* ~ *pair* + *reward* + *PC1_size_* + *PC2_size_* + (*1*|*sp*), where *reward* was either reward presence (*RwP*) or reward type (*RwT*), and *contrast* either chromatic or achromatic contrast. We obtained fixed-effect significance (*F*, *p*) using lmerTest [80], and model fit was summarized with marginal and conditional *R*^2^. These models tested whether chromatic and achromatic contrasts differed consistently among floral structures, whether they scaled with flower and display size, and whether the reward system modulated intrafloral color differentiation once size and structure were accounted for.

## 5. Conclusions

Bee-pollinated orchids exhibit a remarkable diversity in visual signals, exhibiting a spectrum of reward-linked visual strategies structured by intrafloral color modules and color–size integration. In general, rewardless, food-deceptive orchids present achromatically exaggerated visual signals traded off by smaller displays compared to rewarding species, while chromatically mimicking the generalized nectar-rewarding signals of co-flowering species. Rewarding orchids that offer oils or fragrances, in turn, exhibit reduced conspicuousness and may rely more on specialized olfactory cues, including nest-related and courtship-related scents, and larger displays, than on spectral signals for pollination attraction. The absence of variation in intrafloral modularity and in the strength of color–size integration across reward systems shows that both differential organization of floral cues among flower modules and scaling effects with display and flower size contribute to the diversification of orchid visual strategies. Regardless of reward type, orchids with larger flowers presented higher chromatic contrasts, both between intrafloral color modules and against the background, possibly for extra guidance. Species with larger inflorescences presented increasingly lower intra- and extrafloral achromatic contrasts. Future work combining detailed measurements of reward quantity and quality, floral and display size, and visual and olfactory contexts within ecological communities will be essential to clarify how signal reliability and deception evolve under the complex economic and cognitive constraints of bee pollination.

## Figures and Tables

**Table 1:** Loadings of the four composite variables (*PC1_size_*, *PC2_size_*, *PC1_color_*, and *PC2_color_*) on the first four dimensions of the final PCA, which was performed on the principal components extracted separately from floral size (PCA_size_) and color (PCA_color_). Values indicate the contribution and direction of each original PC to the corresponding dimension; higher absolute values denote stronger influence. *PC1_final_* is primarily associated with overall flower size (*PC1_size_*) and chromatic contrast (*PC2_color_*), whereas *PC2_final_* captures achromatic contrast (*PC1_color_*) and display size (*PC2_size_*). Finally, *PC3_final_* and *PC4_final_* capture secondary, orthogonal variation reflecting mixed contributions of size and color components.

Variable	*PC1_final_*	*PC2_final_*	*PC3_final_*	*PC4_final_*
*PC1_size_* (flower size)	0.84	0.17	−0.10	0.51
*PC2_size_* (display size)	0.20	−0.78	0.60	0.07
*PC1_color_* (ACB)	−0.36	0.72	0.55	0.19
*PC2_color_* (CCB)	0.77	0.35	0.21	−0.47

**Table 2:** PERMANOVA testing differences in multivariate visual traits among reward type (*RwT*) and presence (*RwP*). Analyses were conducted on (i) final PCA scores combining color and size (*PC1_final_*, *PC2_final_*), (ii) color PCA scores (*PC1_color_*, *PC2_color_*), (iii) size PCA scores (*PC1_size_*, *PC2_size_*), and (iv) hexagon coordinates (*x*, *y*) of individual floral modules (sepals, petals, lip tip, and lip base) in bee color space. Grouping factors were reward type (*RwT*: unknown, deceit, nectar, oil, and fragrance) or reward presence (*RwP*: unknown, rewardless, rewarding). For each model, the table shows the proportion of variance explained (*R*^2^), pseudo-F statistic (*F*), permutation-based *p*-value (999 permutations), number of observations (*N*), and number of groups compared (groups). Significant *p*-values indicate differences in multivariate centroid location among reward categories.

Variables	Reward	*R* ^2^	*F*	*p*	*N*	Groups
*PC1_final_*, *PC2_final_*	*RwP*	0.11	4.45	0.005	74	3
*PC1_final_*, *PC2_final_*	*RwT*	0.18	3.80	0.002	74	5
*PC1_color_*, *PC2_color_*	*RwP*	0.12	4.97	0.003	74	3
*PC1_color_*, *PC2_color_*	*RwT*	0.15	3.07	0.006	74	5
*PC1_size_*, *PC2_size_*	*RwP*	0.04	2.16	0.076	95	3
*PC1_size_*, *PC2_size_*	*RwT*	0.11	2.79	0.010	95	5
*x_sep_*, *y_sep_*	*RwP*	0.17	8.28	0.001	85	3
*x_sep_*, *y_sep_*	*RwT*	0.17	4.13	0.001	85	5
*x_pet_*, *y_pet_*	*RwP*	0.23	11.08	0.001	79	3
*x_pet_*, *y_pet_*	*RwT*	0.25	6.30	0.001	79	5
*x_lip_api_*, *y_lip_api_*	*RwP*	0.06	2.97	0.030	91	3
*x_lip_api_*, *y_lip_api_*	*RwT*	0.10	2.39	0.035	91	5
*x_lip_bas_*, *y_lip_bas_*	*RwP*	0.06	2.79	0.031	85	3
*x_lip_bas_*, *y_lip_bas_*	*RwT*	0.09	1.93	0.075	85	5

**Table 3:** Phylogenetic signal (Blomberg’s *K*) for principal-component traits in bee-pollinated orchids. Values were computed using the S2 phylogeny built with V.PhyloMaker2 [44]. For each trait, *N* is the number of species, *K* is Blomberg’s *K*, where *K* = 1 indicates Brownian-motion-like evolution; *K* < 1 indicates weaker phylogenetic signal than Brownian expectation, and *K* > 1 indicates stronger clustering among relatives, and *p* is the randomization-test for *K* (null: no phylogenetic signal).

Variable	*N*	*K*	*p*
*PC1_final_*	74	0.47	0.001
*PC2_final_*	74	0.33	0.003
*PC1_color_*	74	0.16	0.553
*PC2_color_*	74	0.41	0.001
*PC1_size_*	95	0.44	0.001
*PC2_size_*	95	0.37	0.001

**Table 4:** Linear regression results relating color and size principal components. For each model, *n* is the number of species, *R*^2^ is the proportion of variance explained, *F* is the model F-statistic, and *p* is the corresponding significance level.

Model	*n*	*R* ^2^	*F*	*p*
*PC1_color_*~*PC1_size_*	74	0.02	1.62	0.20719
*PC1_color_*~*PC2_size_*	74	0.09	6.76	0.01129
*PC2_color_*~*PC1_size_*	*74*	*0.22*	*20.12*	*0.00003*
*PC2_color_*~*PC2_size_*	74	0.00	0.05	0.81930

## Data Availability

All data used in this study are available in the Supplementary Materials.

## Supplementary Materials

The following supporting information can be downloaded at: https://www.mdpi.com/article/10.3390/plants15010154/s1. Figure S1: Heatmaps of standardized residuals from chi-squared tests between reward type and color categories; Table S1: Variable loadings for the three PCA analyses of visual reward differences; Table S2: Pairwise PERMANOVA results identifying group differences in multivariate trait space; Table S3: Permutation tests of multivariate dispersion used to validate PERMANOVA results; Table S4: Phylogenetically informed ANOVAs of principal components of visual traits according to reward systems; Figure S2: Phylogenetically-informed score difference tests of the first two principal components according to reward systems; Figure S3: Scatterplots presenting linear regressions between PCS and mean pollinator size; Figure S4: Boxplots of mean pollinator size comparisons between reward types; Table S5: Results of linear mixed-effects models testing the effects of flower and display size on bee contrasts of floral module pairs; Table S6. Pollinators and mean pollinator body size dataset for bee-pollinated orchids sampled; Table S7: Intertegular span data for 744 bee species compiled from literature.

## Abbreviations

The following abbreviations are used in this manuscript:

PC1	First Principal Component
PC2	Second Principal Component
CCB	Chromatic Contrast against the Background
ACB	Achromatic Contrast against the Background
SP	Spectral Purity
ITS	Intertegular Span
JBRJ	Rio de Janeiro Botanical Garden

## Appendix A

**Table A1:** Species sampled, their PCA scores and their reward systems, extracted from Ackerman et al. (2023) [3]. We also used Ackerman et al. (2023) [3] to check the related species of unstudied species and infer bee-pollination, and to extract pollinator species for mean pollinator size calculations. Reward systems are food deceit (dec), nectar (nec), oil (oil), fragrance (fra), and unknown (unk). *PC1_global_* represents the achromatic dimension of the global PCA, while *PC2_global_* represents the covariance of chromatic and size signals. *PC1_color_* also represents the achromatic dimension of the color PCA, while PC2_color_ shows the chromatic axis. *PC1_size_* represents the flower size dimension of the size PCA, while *PC2_size_* represents the display size.

Species	*RwT*	*PC1_size_*	*PC2_size_*	*PC1_color_*	*PC2_color_*	*PC1_final_*	*PC2_final_*
*Arundina graminifolia* (D.Don) Hochr.	dec	3.45	0.32	1.57	2.91	1.79	0.83
*Bifrenaria inodora* Lindl.	unk	3.36	−0.82	-	-	-	-
*Catasetum cernuum* Rchb.f.	fra	1.20	1.44	−3.31	−2.32	0.18	−1.72
*Catasetum fimbriatum* Lindl. & Paxton	fra	2.26	0.55	−2.34	−2.09	0.41	−0.95
*Catasetum luridum* (Link) Lindl.	fra	1.22	0.72	−0.32	−0.06	0.43	−0.38
*Catasetum multifidum* F.E.L.Miranda	fra	1.19	0.13	−3.57	0.05	0.72	−0.81
*Catasetum pileatum* Rchb.f.	fra	0.22	0.97	−0.82	−1.34	−0.20	−0.87
*Cattleya crispata* (Thunb.) Van den Berg	unk	−1.15	0.06	-	-	-	-
*Cattleya guttata* Lindl.	unk	3.23	0.06	−3.06	0.69	1.56	−0.41
*Cattleya intermedia* Graham ex Hook.	dec	4.43	−1.14	−0.65	4.23	2.57	1.30
*Cattleya labiata* Lindl. var. coerulea	unk	4.90	0.20	0.59	1.49	1.99	0.58
*Cattleya walkeriana* Gardner	unk	4.48	0.07	−0.41	2.23	2.14	0.48
*Chiloschista parishii* Seidenf.	unk	−3.43	−0.52	2.76	−0.67	−1.85	0.48
*Chysis bractescens* Lindl.	unk	2.68	0.14	2.11	−0.62	0.46	0.49
*Coelogyne flaccida* Lindl.	dec	−0.29	0.04	4.55	−1.48	−1.10	0.74
*Coelogyne huettneriana* Rchb.f.	unk	1.00	0.22	-	-	-	-
*Coelogyne viscosa* Rchb.f.	dec	1.22	−1.05	3.16	2.03	0.44	1.56
*Cycnoches haagii* Barb. Rodr.	fra	1.95	0.93	−1.96	−0.36	0.80	−0.83
*Cycnoches pentadactylon* Lindl.	fra	−1.06	0.52	−1.80	−1.29	−0.58	−0.96
*Cymbidium aloifolium* (L.) Sw.	oil	−0.31	0.48	−4.33	−0.43	0.19	−1.33
*Cyrtopodium flavum* (Nees) Link & Otto ex Rchb.	unk	−0.12	2.23	2.89	−1.77	−0.69	−0.73
*Cyrtopodium gigas* (Vell.) Hoehne	unk	−0.45	1.81	−0.22	−2.98	−0.86	−1.42
*Dendrobium anosmum* Lindl.	nec	3.00	−1.07	0.37	2.93	1.61	1.21
*Dendrobium antennatum* Lindl.	unk	0.03	−0.34	3.07	0.81	−0.23	0.94
*Dendrobium aphyllum* (Roxb.) C.E.C.Fisch.	dec	1.79	−4.96	1.19	1.81	0.33	3.04
*Dendrobium convolutum* Rolfe	unk	−0.19	−0.18	−0.02	−3.40	−1.14	−0.44
*Dendrobium cymbidioides* (Blume) Lindl.	unk	−1.38	−0.88	4.14	−1.96	−1.68	0.95
*Dendrobium delacourii* Guillaumin	unk	−1.80	−0.15	1.33	−1.58	−1.33	−0.02
*Dendrobium farmeri* Paxton	unk	0.94	0.11	3.33	2.63	0.63	1.09
*Dendrobium hancockii* Rolfe	nec	0.38	−2.98	2.71	−1.80	−1.11	1.81
*Dendrobium kingianum* Bidwill ex Lindl	nec	−1.35	−0.15	-	-	-	-
*Dendrobium lasianthera* J.J.Sm.	unk	1.35	0.56	−1.23	−2.19	−0.05	−0.79
*Dendrobium lindleyi* Steud.	unk	−0.15	−0.03	0.95	−1.42	−0.66	−0.02
*Dendrobium loddigesii* Rolfe	nec	−1.75	−3.52	-	-	-	-
*Dendrobium nobile* Lindl.	dec	1.36	−2.00	-	-	-	-
*Dendrobium polysema* Schltr.	unk	0.18	0.02	1.52	−0.11	−0.23	0.29
*Dendrobium speciosum* Sm.	dec	−0.74	1.17	4.96	4.77	0.59	1.11
*Dendrobium sulcatum* Lindl.	nec	0.74	−0.80	1.92	−1.87	−0.67	0.59
*Dichaea pendula* (Aubl.) Cogn.	fra	−3.76	−2.48	−3.24	−0.73	−1.57	0.06
*Encyclia alboxanthina* Fowlie	unk	−0.60	1.04	−1.34	−0.89	−0.30	−1.02
*Encyclia argentinensis* (Speg.) Hoehne	unk	0.78	0.81	−0.66	−0.71	0.13	−0.62
*Encyclia cordigera* (Kunth) Dressler	dec	2.35	−0.06	−4.63	1.75	1.70	−0.63
*Encyclia diurna* (Jacq.) Schltr.	unk	−1.19	1.59	-	-	-	-
*Encyclia linearifolioides* (Kraenzl.) Hoehne	unk	−0.89	−0.21	0.66	−0.25	−0.58	0.11
*Encyclia maderoi* Schltr.	unk	−0.73	0.96	−0.67	−2.04	−0.75	−0.99
*Encyclia mapuerae* (Huber) Brade & Pabst	nec	−0.23	0.57	−3.09	0.63	0.39	−0.94
*Encyclia patens* Hook.	nec	−1.04	0.30	−3.98	2.04	0.57	−0.87
*Encyclia replicata* (Lindl. & Paxton) Schltr.	unk	−1.47	3.19	−3.61	−1.78	−0.36	−2.77
*Gomesa ciliata* (Lindl.) M.W.Chase & N.H.Williams	oil	−1.97	0.25	−0.18	−3.08	−1.61	−0.78
*Gomesa echinata* (Barb. Rodr.) M.W.Chase & N.H.Williams	oil	−1.32	0.64	−0.29	−1.82	−0.97	−0.77
*Gomesa flexuosa* (Lodd.) M.W.Chase & N.H.Williams	oil	−4.66	2.34	0.55	−0.77	−1.74	−1.52
*Gomesa imperatoris-maximiliani* (Rchb.f.) M.W.Chase & N.H.Williams	oil	0.83	3.10	-	-	-	-
*Gomesa praetexta* (Rchb.f.) M.W.Chase & N.H.Williams	oil	−1.12	2.33	−4.40	−1.60	−0.20	−2.48
*Gomesa recurva* R.Br.	oil	−3.39	−0.14	-	-	-	-
*Gomesa uniflora* (Booth ex Lindl.) M.W.Chase & N.H.Williams	unk	−1.76	0.41	1.21	−3.34	−1.73	−0.56
*Gongora bufonia* Lindl.	fra	0.11	1.63	-	-	-	-
*Grammatophyllum scriptum* (L.) Blume	unk	−0.07	1.88	2.05	−3.26	−1.04	−0.95
*Ionopsis utricularioides* (Sw.) Lindl.	dec	−4.54	1.15	-	-	-	-
*Laelia undulata* (Lindl.) L.O.Williams	unk	1.34	1.53	−5.13	2.29	1.73	−1.52
*Lockhartia lunifera* (Lindl.) Rchb.f.	oil	−3.64	−1.62	2.93	−3.12	−2.75	0.70
*Maxillaria alba* Lindl.	dec	−1.25	−2.59	4.94	1.30	−1.00	2.42
*Maxillaria chrysantha* Barb. Rodr.	unk	1.56	−0.14	-	-	-	-
*Maxillaria crassifolia* (Lindl.) Rchb.f.	unk	−0.68	−2.41	-	-	-	-
*Maxillaria johannis* Pabst	dec	−0.55	−2.88	2.18	−2.56	−1.58	1.46
*Maxillaria richii* Dodson	unk	−1.37	−1.62	−0.21	−0.19	−0.80	0.58
*Maxillaria rodrigueziana* J.T.Atwood & Mora-Ret.	unk	0.75	−2.63	4.21	−0.20	−0.65	2.22
*Maxillaria tenuifolia* Lindl.	unk	0.17	−3.01	−5.55	−0.81	−0.03	0.08
*Maxillaria variabilis* Bateman ex Lindl.	nec	−3.38	−3.42	1.79	−5.43	−3.40	1.02
*Miltonia clowesii* (Lindl.) Lindl.	unk	1.25	0.25	−2.53	−0.01	0.63	−0.64
*Miltonia cuneata* Lindl.	unk	1.80	1.54	-	-	-	-
*Miltonia moreliana* hort. ex Lindl.	unk	2.24	0.24	−2.50	2.15	1.58	−0.25
*Miltonia regnellii* Rchb.f.	unk	1.19	0.21	2.50	−0.54	−0.08	0.44
*Miltonia spectabilis* Lindl.	unk	2.32	−0.28	4.98	1.77	0.64	1.64
*Mormodes morenoi* R.Vásquez & Dodson	fra	1.84	0.19	−6.30	−0.01	1.23	−1.41
*Myrmecophila tibicinis* (Bateman) Rolfe	dec	2.51	1.20	−0.29	1.74	1.44	−0.26
*Notylia lyrata* S.Moore	fra	−6.24	0.84	-	-	-	-
*Papilionanthe teres* Schltr.	unk	2.64	0.01	0.80	3.38	1.69	0.81
*Phalaenopsis deliciosa* Rchb.f.	unk	−3.93	−0.69	-	-	-	-
*Phalaenopsis hieroglyphica* (Rchb.f.) H.R.Sweet	unk	0.48	−0.84	0.13	0.55	0.11	0.51
*Polystachya neobenthamia* Schltr.	unk	−1.69	−0.08	3.04	6.44	0.78	1.45
*Polystachya pubescens* (Lindl.) Rchb.f.	unk	−3.70	−0.01	-	-	-	-
*Prosthechea bulbosa* (Vell.) W.E.Higgins	unk	−1.39	−0.02	4.45	1.28	−0.71	1.05
*Prosthechea radiata* (Lindl.) W.E.Higgins	unk	−1.15	−0.15	4.44	−0.34	−1.09	0.90
*Prosthechea vespa* (Vell.) W.E.Higgins	dec	−1.99	−0.37	-	-	-	-
*Pseudolaelia corcovadensis* Porto & Brade	dec	−0.16	0.80	-	-	-	-
*Sobralia yauaperyensis* Barb. Rodr.	unk	4.26	−0.90	0.58	5.76	2.84	1.66
*Spathoglottis unguiculata* (Labill.) Rchb.f.	dec	−0.96	0.22	−2.03	2.14	0.40	−0.38
*Tolumnia variegata* (Sw.) Braem	dec	−4.41	0.80	-	-	-	-
*Trichocentrum cepula* (Hoffmanns.) J.M.H.Shaw	oil	−0.94	0.96	−1.15	−2.29	−0.84	−1.15
*Trichocentrum jonesianum* (Rchb.f.) M.W.Chase & N.H.Williams	oil	−1.12	0.95	−2.49	1.61	0.34	−0.92
*Trichocentrum lanceanum* (Lindl.) M.W.Chase & N.H.Williams	unk	0.73	1.13	−5.45	4.25	2.06	−1.17
*Vanda tricolor* Lindl.	dec	2.13	0.86	−0.62	−0.01	0.81	−0.43
*Warczewiczella wailesiana* (Lindl.) É.Morren	unk	0.41	−0.16	0.76	1.15	0.27	0.40
*Warrea warreana* (Lodd. ex Lindl.) C.Schweinf.	dec	1.09	−0.07	−0.96	−1.31	0.00	−0.32
*Zygopetalum maculatum* (Kunth) Garay	dec	2.10	0.74	-	-	-	-

**Table A2:** Summary (mean ± standard deviation, sample size) of the variables used in the test of visual reward syndromes in bee-pollinated orchids. Columns indicate different resource types: *deceit* = food deception, *nectar* = nectar, *oil* = floral oils, and *fragrance* = fragrances. Variables: *PC1_global_* and *PC2_glo_* are the principal components from the PCA, including all variables, while *PC1_col_* and *PC2_col_* refer to the PCA including only color variables, and *PC1_size_* and *PC2_size_* to that including only size variables. *Depth* = floral depth; *radius* = floral radius; *length_sep_* = sepal length; *length_pet_* = petal length; *length_lip_* = labellum length; *N_flo_*_/_*_inf_* = number of flowers per inflorescence; *N_inf_*_/_*_ind_* = number of inflorescences per individual; *ACB* = achromatic contrast against the background; *CCB* = chromatic contrast against the background; *SP* = spectral purity; *sep* = sepals; *pet* = petals; *lip_api* = apex of the labellum; *lip_bas* = base of the labellum; *UV*, *blue*, *green*, and *red* = reflectance intensities in the ultraviolet (300–400 nm), blue (400–500 nm), green (500–600 nm), and red (600–700 nm) wavebands; *N_pollinator_* = number of known pollinator species; *ITS* = mean intertegular distance of the known pollinator species.

Variable	Deceit	Nectar	Oil	Fragrance
*PC1_final_*	0.49 ± 1.22 (*n* = 13)	−0.44 ± 1.74 (*n* = 6)	−0.95 ± 1.06 (*n* = 8)	0.16 ± 0.84 (*n* = 9)
*PC2_final_*	0.80 ± 1.17 (*n* = 13)	0.47 ± 1.14 (*n* = 6)	−1.03 ± 0.89 (*n* = 8)	−0.87 ± 0.52 (*n* = 9)
*PC1_color_*	1.03 ± 2.91 (*n* = 13)	−0.05 ± 2.82 (*n* = 6)	−1.17 ± 2.50 (*n* = 8)	−2.63 ± 1.77 (*n* = 9)
*PC2_color_*	1.33 ± 2.17 (*n* = 13)	−0.58 ± 3.08 (*n* = 6)	−1.44 ± 1.57 (*n* = 8)	−0.91 ± 0.90 (*n* = 9)
*PC1_size_*	0.40 ± 2.42 (*n* = 19)	−0.45 ± 1.92 (*n* = 8)	−1.77 ± 1.68 (*n* = 10)	−0.10 ± 2.68 (*n* = 11)
*PC2_size_*	−0.41 ± 1.67 (*n* = 19)	−1.39 ± 1.69 (*n* = 8)	0.93 ± 1.38 (*n* = 10)	0.50 ± 1.09 (*n* = 11)
*depth* (mm)	17.41 ± 12.87 (*n* = 19)	14.35 ± 9.41 (*n* = 8)	3.86 ± 4.68 (*n* = 10)	6.45 ± 9.48 (*n* = 11)
*radius* (mm)	23.86 ± 12.41 (*n* = 19)	17.74 ± 9.02 (*n* = 8)	14.42 ± 6.78 (*n* = 10)	23.90 ± 10.77 (*n* = 11)
*length_sep_* (mm)	24.00 ± 11.68 (*n* = 19)	18.89 ± 10.03 (*n* = 8)	15.34 ± 8.80 (*n* = 10)	25.99 ± 12.43 (*n* = 11)
*length_pet_* (mm)	23.81 ± 12.75 (*n* = 19)	18.54 ± 11.83 (*n* = 8)	12.43 ± 6.40 (*n* = 10)	24.14 ± 11.86 (*n* = 11)
*length_lip_* (mm)	23.83 ± 12.74 (*n* = 19)	16.44 ± 8.17 (*n* = 8)	14.95 ± 7.98 (*n* = 10)	22.79 ± 12.73 (*n* = 11)
*area* (mm^2^)	810 ± 915 (*n* = 19)	461 ± 425 (*n* = 8)	459 ± 428 (*n* = 10)	674 ± 620 (*n* = 11)
*length_inf_* (mm)	294 ± 295 (*n* = 19)	117 ± 143 (*n* = 8)	441 ± 233 (*n* = 10)	273 ± 258 (*n* = 11)
*N_flo_*/*_inf_*	8.95 ± 11.63 (*n* = 19)	4.88 ± 3.56 (*n* = 8)	20.00 ± 20.14 (*n* = 10)	12.74 ± 11.90 (*n* = 11)
*N_inf_*/*_ind_*	1.72 ± 1.13 (*n* = 18)	2.00 ± 1.51 (*n* = 8)	1.10 ± 0.32 (*n* = 10)	1.18 ± 0.40 (*n* = 11)
*ACB_lip_api_*	0.15 ± 0.09 (*n* = 19)	0.18 ± 0.07 (*n* = 8)	0.15 ± 0.11 (*n* = 10)	0.12 ± 0.06 (*n* = 10)
*ACB_lip_bas_*	0.14 ± 0.08 (*n* = 16)	0.09 ± 0.09 (*n* = 7)	0.16 ± 0.06 (*n* = 9)	0.10 ± 0.06 (*n* = 10)
*ACB_pet_*	0.19 ± 0.08 (*n* = 15)	0.17 ± 0.05 (*n* = 6)	0.11 ± 0.07 (*n* = 9)	0.10 ± 0.06 (*n* = 9)
*ACB_sep_*	0.17 ± 0.08 (*n* = 16)	0.16 ± 0.08 (*n* = 6)	0.12 ± 0.09 (*n* = 9)	0.10 ± 0.06 (*n* = 11)
*CCB_lip_api_*	0.47 ± 0.16 (*n* = 19)	0.42 ± 0.18 (*n* = 8)	0.43 ± 0.19 (*n* = 10)	0.41 ± 0.12 (*n* = 10)
*CCB_lip_api_bas_*	0.33 ± 0.25 (*n* = 16)	0.26 ± 0.29 (*n* = 7)	0.40 ± 0.23 (*n* = 9)	0.30 ± 0.27 (*n* = 10)
*CCB_lip_bas_*	0.44 ± 0.20 (*n* = 16)	0.47 ± 0.28 (*n* = 7)	0.33 ± 0.13 (*n* = 9)	0.46 ± 0.17 (*n* = 10)
*CCB_pet_*	0.44 ± 0.13 (*n* = 15)	0.32 ± 0.15 (*n* = 6)	0.37 ± 0.13 (*n* = 9)	0.47 ± 0.15 (*n* = 9)
*CCB_pet_lip_api_*	0.41 ± 0.23 (*n* = 15)	0.36 ± 0.24 (*n* = 6)	0.33 ± 0.28 (*n* = 9)	0.37 ± 0.20 (*n* = 9)
*CCB_pet_lip_bas_*	0.31 ± 0.20 (*n* = 14)	0.48 ± 0.27 (*n* = 6)	0.36 ± 0.20 (*n* = 8)	0.35 ± 0.23 (*n* = 9)
*CCB_sep_*	0.43 ± 0.11 (*n* = 16)	0.38 ± 0.18 (*n* = 6)	0.37 ± 0.12 (*n* = 9)	0.44 ± 0.13 (*n* = 11)
*CCB_sep_lip_api_*	0.38 ± 0.21 (*n* = 16)	0.37 ± 0.30 (*n* = 6)	0.34 ± 0.26 (*n* = 9)	0.32 ± 0.24 (*n* = 10)
*CCB_sep_lip_bas_*	0.32 ± 0.21 (*n* = 15)	0.50 ± 0.28 (*n* = 6)	0.33 ± 0.12 (*n* = 8)	0.29 ± 0.25 (*n* = 10)
*CCB_sep_pet_*	0.09 ± 0.05 (*n* = 15)	0.13 ± 0.10 (*n* = 6)	0.19 ± 0.12 (*n* = 9)	0.14 ± 0.10 (*n* = 9)
*SP_lip_api_*	0.43 ± 0.22 (*n* = 19)	0.30 ± 0.17 (*n* = 8)	0.24 ± 0.20 (*n* = 10)	0.38 ± 0.15 (*n* = 10)
*SP_lip_bas_*	0.43 ± 0.23 (*n* = 16)	0.33 ± 0.20 (*n* = 7)	0.28 ± 0.13 (*n* = 9)	0.40 ± 0.13 (*n* = 10)
*SP_pet_*	0.50 ± 0.24 (*n* = 15)	0.23 ± 0.17 (*n* = 6)	0.21 ± 0.11 (*n* = 9)	0.23 ± 0.10 (*n* = 9)
*SP_sep_*	0.44 ± 0.23 (*n* = 16)	0.20 ± 0.17 (*n* = 6)	0.22 ± 0.09 (*n* = 9)	0.27 ± 0.19 (*n* = 11)
*blue_lip_api_*	0.40 ± 0.26 (*n* = 19)	0.37 ± 0.32 (*n* = 8)	0.13 ± 0.23 (*n* = 10)	0.19 ± 0.09 (*n* = 10)
*blue_lip_bas_*	0.37 ± 0.25 (*n* = 16)	0.10 ± 0.12 (*n* = 7)	0.07 ± 0.07 (*n* = 9)	0.16 ± 0.13 (*n* = 10)
*blue_pet_*	0.50 ± 0.27 (*n* = 15)	0.21 ± 0.26 (*n* = 6)	0.07 ± 0.06 (*n* = 9)	0.12 ± 0.09 (*n* = 9)
*blue_sep_*	0.48 ± 0.25 (*n* = 16)	0.21 ± 0.26 (*n* = 6)	0.09 ± 0.08 (*n* = 9)	0.10 ± 0.08 (*n* = 11)
*green_lip_api_*	0.47 ± 0.33 (*n* = 19)	0.55 ± 0.26 (*n* = 8)	0.54 ± 0.41 (*n* = 10)	0.38 ± 0.21 (*n* = 10)
*green_lip_bas_*	0.43 ± 0.33 (*n* = 16)	0.31 ± 0.33 (*n* = 7)	0.54 ± 0.22 (*n* = 9)	0.33 ± 0.22 (*n* = 10)
*green_pet_*	0.58 ± 0.31 (*n* = 15)	0.58 ± 0.24 (*n* = 6)	0.37 ± 0.27 (*n* = 9)	0.30 ± 0.21 (*n* = 9)
*green_sep_*	0.54 ± 0.31 (*n* = 16)	0.54 ± 0.28 (*n* = 6)	0.40 ± 0.32 (*n* = 9)	0.32 ± 0.20 (*n* = 11)
*red_lip_api_*	0.79 ± 0.15 (*n* = 19)	0.87 ± 0.10 (*n* = 8)	0.65 ± 0.33 (*n* = 10)	0.49 ± 0.11 (*n* = 10)
*red_lip_bas_*	0.79 ± 0.13 (*n* = 16)	0.71 ± 0.24 (*n* = 7)	0.82 ± 0.16 (*n* = 9)	0.49 ± 0.13 (*n* = 10)
*red_pet_*	0.76 ± 0.18 (*n* = 15)	0.73 ± 0.24 (*n* = 6)	0.57 ± 0.17 (*n* = 9)	0.42 ± 0.09 (*n* = 9)
*red_sep_*	0.70 ± 0.21 (*n* = 16)	0.67 ± 0.32 (*n* = 6)	0.54 ± 0.20 (*n* = 9)	0.42 ± 0.09 (*n* = 11)
*UV_lip_api_*	0.05 ± 0.06 (*n* = 19)	0.07 ± 0.04 (*n* = 8)	0.16 ± 0.13 (*n* = 10)	0.05 ± 0.04 (*n* = 10)
*UV_lip_bas_*	0.10 ± 0.14 (*n* = 16)	0.05 ± 0.04 (*n* = 7)	0.11 ± 0.15 (*n* = 9)	0.09 ± 0.10 (*n* = 10)
*UV_pet_*	0.07 ± 0.08 (*n* = 15)	0.13 ± 0.15 (*n* = 6)	0.06 ± 0.11 (*n* = 9)	0.10 ± 0.11 (*n* = 9)
*UV_sep_*	0.07 ± 0.08 (*n* = 16)	0.13 ± 0.11 (*n* = 6)	0.06 ± 0.09 (*n* = 9)	0.06 ± 0.07 (*n* = 11)
*N_pollinator_*	2.43 ± 2.38 (*n* = 14)	1.80 ± 0.84 (*n* = 5)	1.00 ± 0.00 (*n* = 4)	2.00 ± 2.06 (*n* = 9)
*ITS* (mm)	4.29 ± 1.44 (*n* = 14)	3.49 ± 1.00 (*n* = 4)	3.37 ± 1.27 (*n* = 4)	4.39 ± 1.16 (*n* = 8)
