# Supplementary material for: Colors for Resources: Reward-Linked Visual Displays in Orchids
Source: Plants (Basel). 2026 Jan 4;15(1):154. doi: 10.3390/plants15010154 (PMC12787614; doi:10.3390/plants15010154)
Supplement: Supplementary file 1 [file plants-15-00154-s001.zip › Figures S1-S5 and Tables S1-S7.pdf]

plants MDPI

Supplementary Materials

Colors for Resources: Reward-Linked Visual Displays in Orchids

Gabriel Coimbra, Carlos E. Pereira Nunes, Pedro J. Bergamo, João M. R. B. V. Aguiar, Leandro Freitas

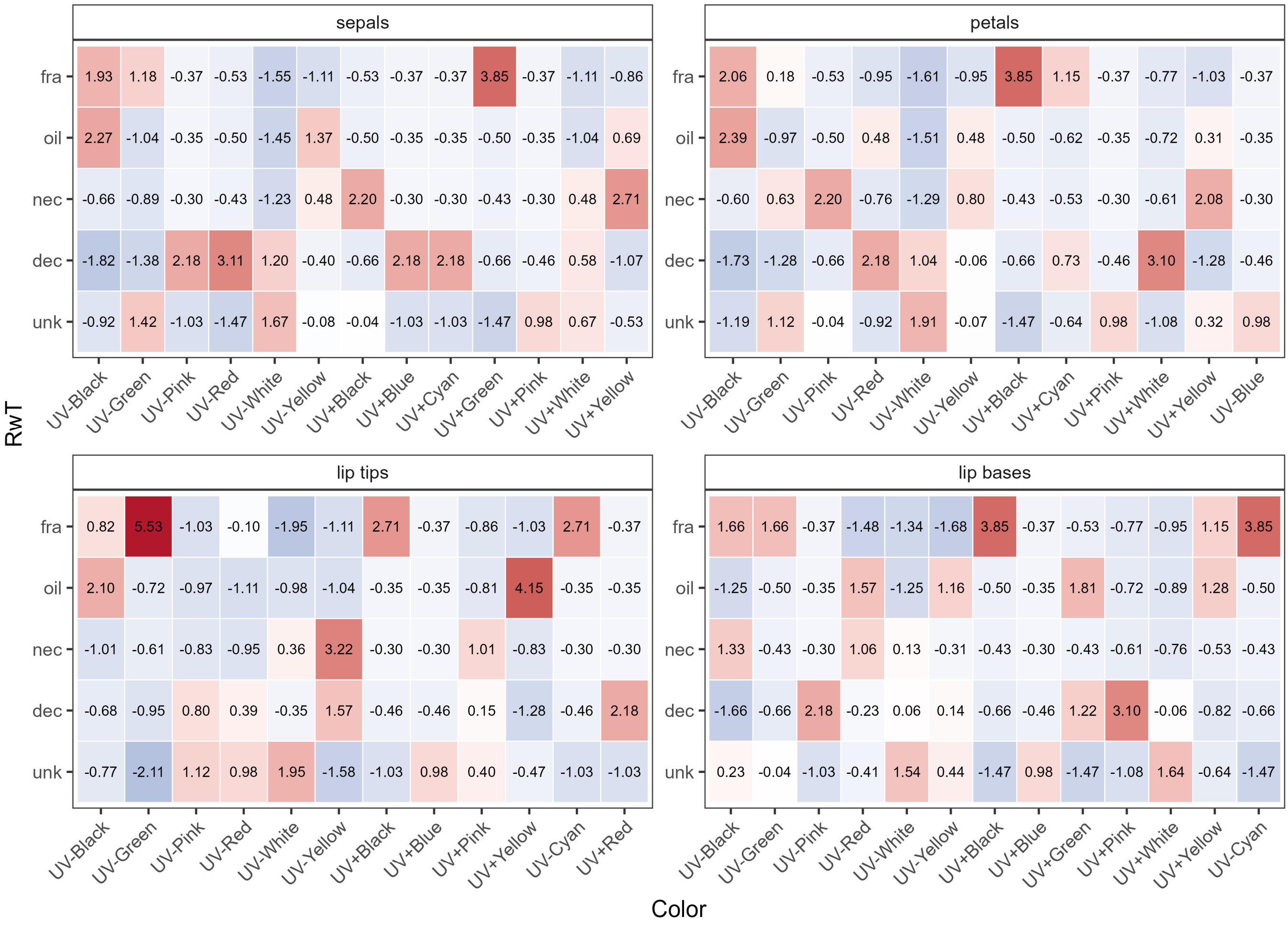

Figure S1. Heatmaps of standardized residuals from chi-squared tests between reward type (RwT) and color categories across four floral modules of bee-pollinated orchids: sepals, petals, lip tips, and lip bases. For each module, a Pearson chi-squared test with simulated p-values ($1{\times}10^6$ replicates) was applied, revealing significant associations in all cases. Darker red tones indicate RwT–color combinations occurring more frequently than expected (positive residuals), while darker blue tones indicate combinations occurring less frequently than expected (negative residuals).

Table S1: Loadings for the variables of the global PCA analysis used in the test of visual reward differences in bee-pollinated orchids. Columns: *PC1glo* and *PC2glo* are the first dimensions of the global PCA. *Depth* = floral depth; *radius* = floral radius; $length_{sep}$ = sepal length; $length_{pet}$ = petal length; $length_{lip}$ = labellum length; $N_{flo/inf}$ = number of flowers per inflorescence; $N_{inf/ind}$ = number of inflorescences per individual; *ACB* = achromatic contrast against the background; *CCB* = chromatic contrast against the background; *SP* = spectral purity; *sep* = sepals; *pet* = petals; *lip_api* = apex of the labellum; *lip_bas* = base of the labellum; *UV, blue, green,* and *red* = reflectance intensities in the ultraviolet (300–400 nm), *blue* (400–500 nm), green (500–600 nm), and *red* (600–700 nm) wavebands.

variable	PC_global	PC2_global	PC1_color	PC2_color	PC1_size	PC1_size
ACB_{lip_api}	0.56	-0.01	0.52	-0.05	-	-
ACB_{lip_bas}	0.45	0.14	0.52	0.05	-	-
ACB_{pet}	0.81	0.35	0.88	-0.01	-	-
ACB_{sep}	0.80	0.44	0.90	0.06	-	-
$area_{flo}$ (mm)	-0.52	0.56	-	-	0.90	0.21
$blue_{lip\_api}$	-0.06	0.42	0.11	0.57	-	-
$blue_{lip\_bas}$	0.06	0.28	0.18	0.31	-	-
$blue_{pet}$	0.27	0.84	0.57	0.61	-	-
$blue_{sep}$	0.30	0.84	0.59	0.60	-	-
CCB_{lip_api}	-0.48	0.37	-0.31	0.60	-	-
$CCB_{lip\_api\_bas}$	-0.14	0.17	-0.07	0.14	-	-
CCB_{lip_bas}	-0.25	0.27	-0.16	0.39	-	-
CCB_{pet}	-0.37	0.42	-0.19	0.66	-	-
$CCB_{pet\_lip\_api}$	-0.45	0.11	-0.38	0.28	-	-
$CCB_{pet\_lip\_bas}$	-0.52	0.26	-0.38	0.39	-	-
CCB_{sep}	-0.52	0.24	-0.41	0.48	-	-
$CCB_{sep\_lip\_api}$	-0.48	0.18	-0.40	0.48	-	-
$CCB_{sep\_lip\_bas}$	-0.51	0.18	-0.43	0.45	-	-
CCB_{sep_pet}	-0.27	-0.02	-0.28	0.30	-	-
$depth_{flo}$	-0.12	0.58	-	-	0.52	-0.36
$green_{lip\_api}$	0.60	-0.09	0.52	-0.14	-	-
$green_{lip\_bas}$	0.48	0.09	0.53	0.00	-	-
$green_{pet}$	0.83	0.25	0.85	-0.10	-	-
$green_{sep}$	0.84	0.35	0.90	-0.02	-	-
$length_{inf}$	-0.26	-0.29	-	-	-0.11	0.86
$length_{lip}$	-0.51	0.59	-	-	0.91	0.11
$N_{flo/inf}$	-0.04	-0.33	-	-	-0.34	0.64
Ninf/ind	0.24	0.09	-	-	0.01	-0.80
$length_{pet}$	-0.47	0.65	-	-	0.95	0.08
$radius_{flo}$	-0.52	0.65	-	-	0.98	0.10
red_{lip_api}	0.45	0.18	0.48	0.13	-	-
red_{lip_bas}	0.41	0.32	0.53	0.27	-	-
red_{pet}	0.67	0.50	0.80	0.18	-	-
red_{sep}	0.66	0.54	0.80	0.19	-	-

$length_{sep}$	-0.48	0.59	-	-	0.92	0.04
SP_{lip_api}	-0.33	0.35	-0.18	0.63	-	-
SP_{lip_bas}	-0.24	0.31	-0.11	0.57	-	-
SP_{pet}	0.03	0.69	0.28	0.77	-	-
SP_{sep}	-0.08	0.63	0.15	0.69	-	-
UV_{lip_api}	0.10	-0.16	0.04	-0.20	-	-
UV_{lip_bas}	-0.09	0.14	-0.02	0.03	-	-
UV_{pet}	0.17	0.14	0.20	-0.12	-	-
UV_{sep}	0.19	0.22	0.25	-0.02	-	-

Table S2: Pairwise PERMANOVA results identifying group differences in multivariate trait space. The table summarizes significant pairwise comparisons derived from PERMANOVA models for each response space (final PCA, color PCA, size PCA, and floral-module hexagon coordinates) under the grouping factors reward presence (RwP) and reward type (RwT). For each comparison, we report the two contrasted groups (group1, group2), their respective sample sizes (n1, n2), pseudo-F statistics (F), the proportion of explained variance (R^2), and permutation-based p-values (p). Only pairwise contrasts with $p \leq 0.05$ are shown. These results indicate which specific reward categories differ in multivariate centroid location after accounting for overall group effects, revealing that most significant differences are associated with contrasts involving oil- and nectar-related strategies versus deceptive or unknown categories, as well as strong separation between reward-present and reward-absent species. Together, these pairwise tests clarify the sources of the global PERMANOVA effects by pinpointing which group combinations drive multivariate divergence across color, size, and intrafloral color-space representations.

**Variables**	**Group**	**group1**	**group2**	**n1**	**n2**	***F***	***R2***	***p***
$PC1_{final}$, $PC2_{final}$	RwP	rwd-	rwd+	13	23	8.27	0.20	0.00
$PC1_{final}$, $PC2_{final}$	RwP	unk	rwd+	38	23	3.55	0.06	0.04
$PC1_{final}$, $PC2_{final}$	RwT	dec	oil	13	8	10.75	0.36	0.00
$PC1_{final}$, $PC2_{final}$	RwT	unk	oil	38	8	6.22	0.12	0.00
$PC1_{final}$, $PC2_{final}$	RwT	dec	fra	13	9	7.41	0.27	0.00
$PC1_{final}$, $PC2_{final}$	RwT	oil	fra	8	9	3.74	0.20	0.05
$PC1_{final}$, $PC2_{final}$	RwT	nec	fra	6	9	3.43	0.21	0.05
$PC1_{color}$, $PC2_{color}$	RwP	rwd-	rwd+	13	23	8.96	0.21	0.00
$PC1_{color}$, $PC2_{color}$	RwP	unk	rwd+	38	23	6.36	0.10	0.01
$PC1_{color}$, $PC2_{color}$	RwT	dec	fra	13	9	10.30	0.34	0.00
$PC1_{color}$, $PC2_{color}$	RwT	unk	fra	38	9	6.97	0.13	0.00
$PC1_{color}$, $PC2_{color}$	RwT	dec	oil	13	8	5.37	0.22	0.01
$PC1_{size}$, $PC2_{size}$	RwT	nec	oil	8	10	5.75	0.26	0.00
$PC1_{size}$, $PC2_{size}$	RwT	unk	oil	47	10	7.88	0.13	0.00
$PC1_{size}$, $PC2_{size}$	RwT	dec	oil	19	10	5.81	0.18	0.01
$PC1_{size}$, $PC2_{size}$	RwT	unk	nec	47	8	3.43	0.06	0.03
x_{sep}, y_{sep}	RwP	rwd-	rwd+	16	26	17.65	0.31	0.00
x_{sep}, y_{sep}	RwP	unk	rwd+	43	26	5.88	0.08	0.01
x_{sep}, y_{sep}	RwP	unk	rwd-	43	16	5.35	0.09	0.02
x_{sep}, y_{sep}	RwT	dec	nec	16	6	9.25	0.32	0.00
x_{sep}, y_{sep}	RwT	dec	oil	16	9	9.23	0.29	0.00
x_{sep}, y_{sep}	RwT	dec	fra	16	11	12.02	0.32	0.00
x_{sep}, y_{sep}	RwT	unk	dec	43	16	5.35	0.09	0.02
x_{sep}, y_{sep}	RwT	unk	fra	43	11	3.72	0.07	0.05
x_{pet}, y_{pet}	RwP	unk	rwd+	40	24	12.36	0.17	0.00

x_{pet}, y_{pet}	RwP	rwd-	rwd+	15	24	22.86	0.38	0.00
x_{pet}, y_{pet}	RwT	unk	fra	40	9	10.45	0.18	0.00
x_{pet}, y_{pet}	RwT	dec	fra	15	9	20.94	0.49	0.00
x_{pet}, y_{pet}	RwT	dec	oil	15	9	12.62	0.36	0.00
x_{pet}, y_{pet}	RwT	dec	nec	15	6	6.20	0.25	0.02
x_{pet}, y_{pet}	RwT	unk	oil	40	9	5.55	0.11	0.02
x_{lip_api}, y_{lip_api}	RwP	rwd-	rwd+	19	28	4.51	0.09	0.02
x_{lip_api}, y_{lip_api}	RwP	unk	rwd+	44	28	4.41	0.06	0.03
x_{lip_api}, y_{lip_api}	RwT	unk	oil	44	10	7.39	0.12	0.00
x_{lip_api}, y_{lip_api}	RwT	dec	oil	19	10	7.84	0.23	0.00
x_{lip_bas}, y_{lip_bas}	RwP	rwd-	rwd+	16	26	3.85	0.09	0.04
x_{lip_bas}, y_{lip_bas}	RwP	unk	rwd+	43	26	3.39	0.05	0.04
x_{lip_bas}, y_{lip_bas}	RwT	dec	oil	16	9	3.46	0.13	0.05

Table S3: Permutation tests of multivariate dispersion (PERMDISP) used to validate PERMANOVA results**.** For each response space (PCA_{final}, PCA_{color}, PCA_{size}, and floral-module hexagon coordinates) and grouping factor (reward presence, RwP; reward type, RwT), we tested for differences in within-group multivariate dispersion using permutation tests on betadisper residuals (permutest, 999 permutations). The table reports degrees of freedom (Df), pseudo-F statistics (F), and permutation-based p-values (p). In all cases, dispersion did not differ significantly among groups (all $p > 0.05$), indicating homogeneity of variances in multivariate space. These results support the validity of the corresponding PERMANOVA inferences, confirming that detected group differences (when present) are attributable to shifts in centroid location rather than differences in within-group dispersion.

Variables	Group	Df	*F*	*p*
$PC1_{final}$, $PC2_{final}$	RwP	2	0.26	0.79
$PC1_{color}$, $PC2_{color}$	RwP	2	0.68	0.51
$PC1_{size}$, $PC2_{size}$	RwP	2	1.16	0.33
$PC1_{final}$, $PC2_{final}$	RwT	4	2.13	0.09
$PC1_{color}$, $PC2_{color}$	RwT	4	2.05	0.10
$PC1_{size}$, $PC2_{size}$	RwT	4	0.74	0.55
x_{sep}, y_{sep}	RwP	2	0.16	0.84
x_{pet}, y_{pet}	RwP	2	0.90	0.41
x_{lip_api}, y_{lip_api}	RwP	2	0.01	1.00
x_{lip_api}, y_{lip_bas}	RwP	2	0.97	0.38
x_{sep}, y_{sep}	RwT	4	0.25	0.91
x_{pet}, y_{pet}	RwT	4	1.02	0.40
x_{lip_api}, y_{lip_api}	RwT	4	0.92	0.44
x_{lip_api}, y_{lip_api}	RwT	4	0.61	0.66

Table S4: Phylogenetically informed ANOVAs (phylANOVA; 5,000 permutations) testing for differences among reward systems based on pollination reward presence (RwP) and reward type (RwT) for the first two axes of the final PCA ($PC1_{final}$, $PC2_{final}$) and of the color PCA ($PC1_{color}$, $PC2_{color}$). Reported values are the F statistic and permutation-based p-values. Significant effects indicate trait differentiation among reward groups after accounting for phylogenetic relatedness

variable	group	*F*	*p*
$PC1_{final}$	RwP	2.25	0.36
$PC2_{final}$	RwP	7.38	0.03
$PC1_{final}$	RwT	2.06	0.54
$PC2_{final}$	RwT	6.28	0.10
$PC1_{color}$	RwP	4.94	0.11
$PC2_{color}$	RwP	5.02	0.10

$PC1_{color}$	RwT	3.39	0.32
$PC2_{color}$	RwT	2.60	0.46
$PC1_{color}$	RwP	2.68	0.28
$PC2_{color}$	RwP	0.99	0.62
$PC1_{color}$	RwT	2.22	0.57
$PC2_{color}$	RwT	4.24	0.26

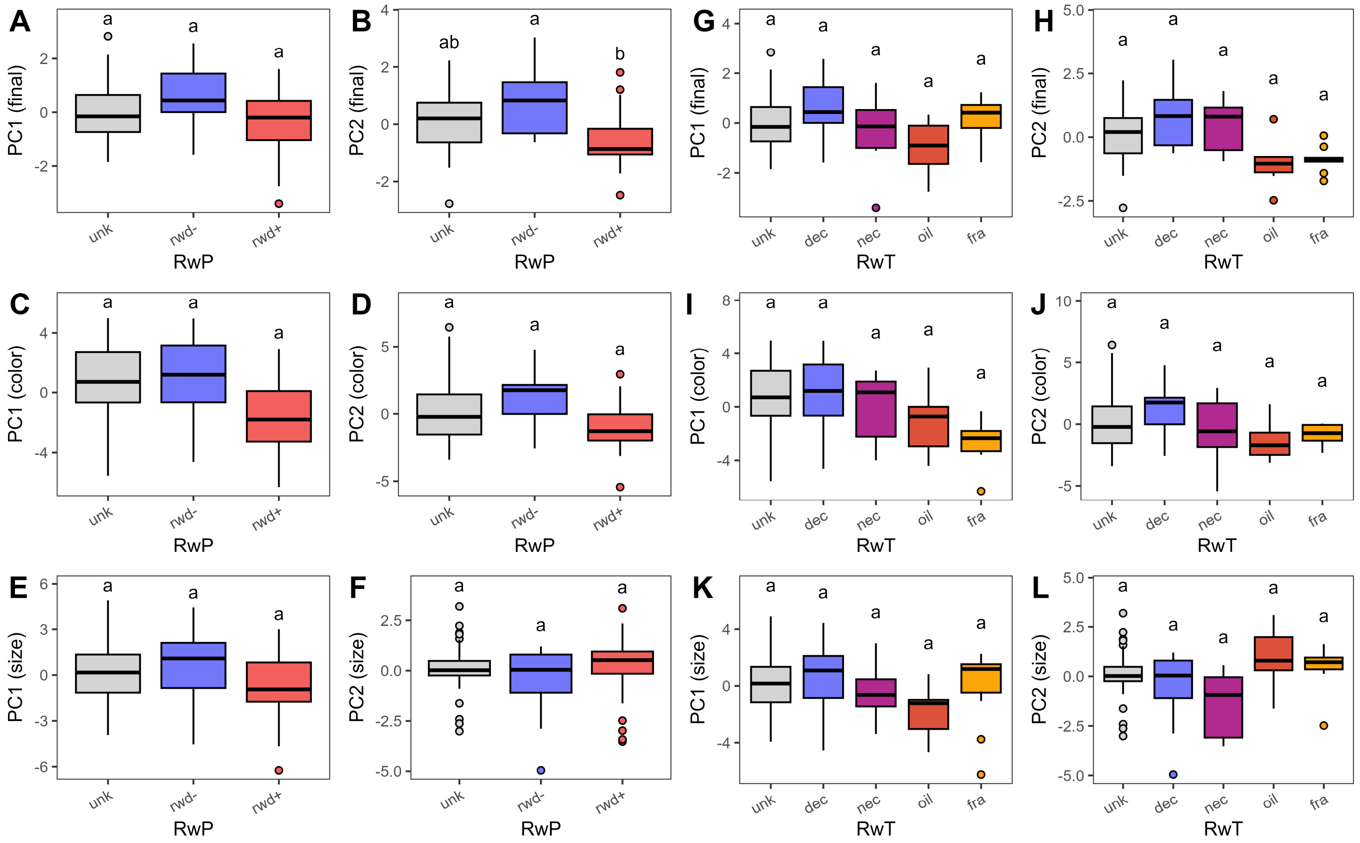

Figure S2. Phylogenetically-informed score difference tests of the first two principal components (PC1 and PC2) of floral traits in bee-pollinated orchids according to reward presence (*RwP*) and type (*RwT*). On the left (A–F), groups include species with unknown reward (unk), without reward (*rwd-*), and with reward (*rwd+*). On the right (G–L), species are classified according to the type of reward offered: deception (*dec*), nectar (*nec*), oil (*oil*), and fragrance (*fra*). Only the second axis of the final PCA ($PC2_{final}$) showed significant differences according to reward presence. Different letters indicate statistically distinct groups according to phylogenetic ANOVAs.

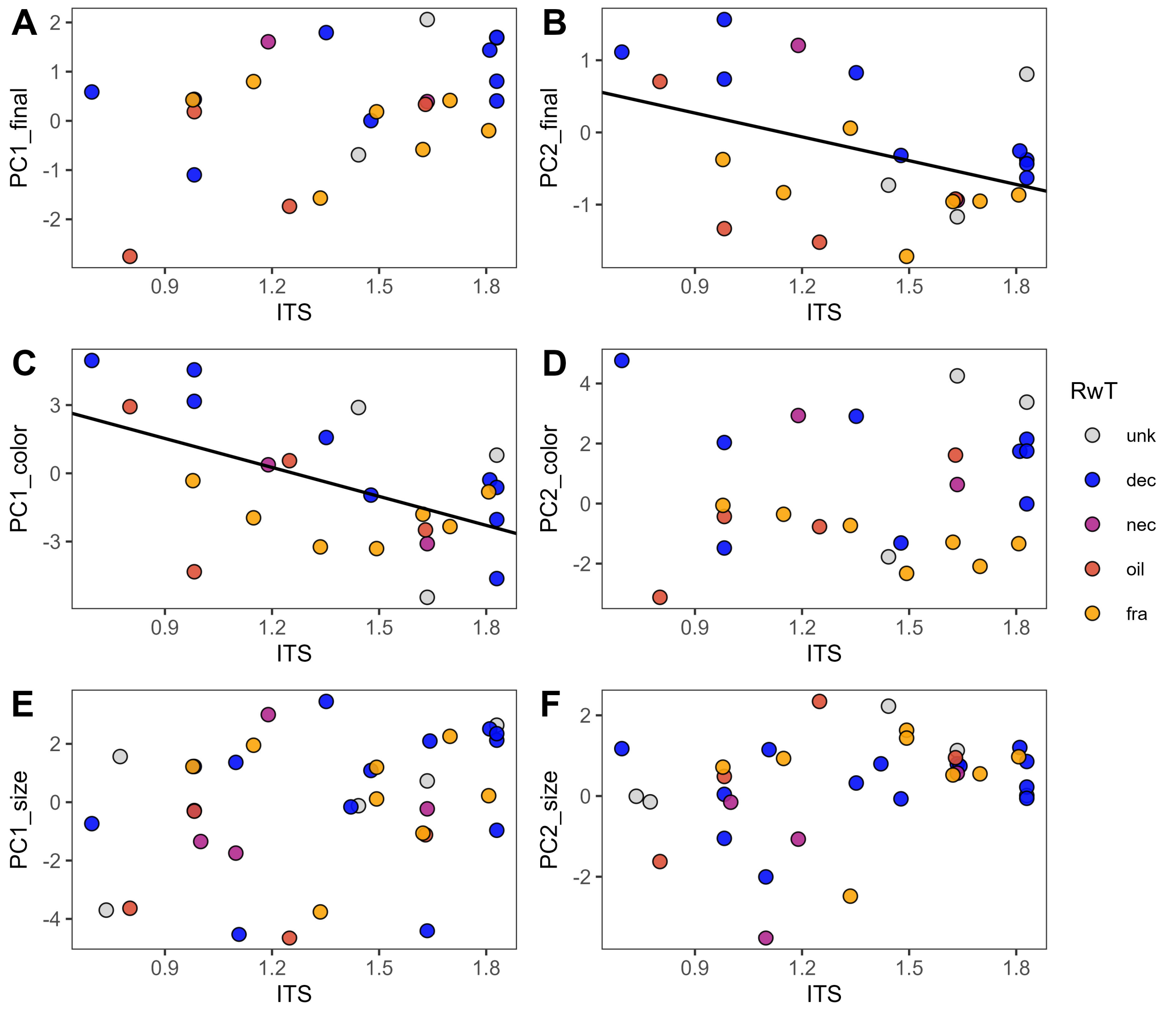

Figure S3: Linear regressions between PCS and mean pollinator size (bee intertegular distances).

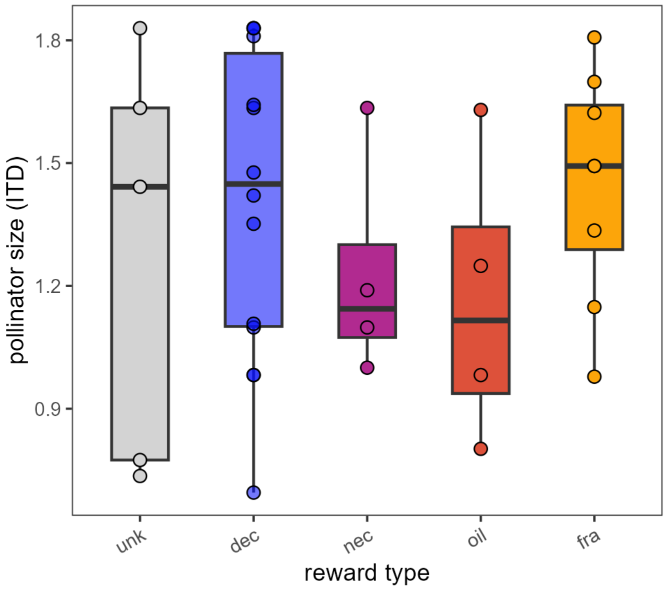

Figure S4: Mean pollinator size (bee intertegular distances) comparisons between reward types.

Table S5: Results of linear mixed-effects models testing the effects of flower and display size on color contrast (CCB_bombus) and achromatic contrast (ACB_bombus) of floral module pairs in bee-pollinated orchids. Models included pair (flower modules compared), reward category (RwP: reward presence; RwT: reward type), and floral size axes (PC1_size representing flower size and PC2_size representing display size) as fixed effects, with species identity (sp) included as a random intercept to account for repeated measurements within species. Reported statistics are F-tests from Type III ANOVA on mixed models, with numerator (NumDF) and denominator (DenDF) degrees of freedom estimated via Satterthwaite's approximation. Significant effects indicate that intrafloral contrasts vary among structure pairs and/or along size axes, while non-significant reward terms indicate no consistent differences in contrast attributable to reward category after accounting for floral size and species-level variation.

**model**	**term**	**NumDF**	**DenDF**	***F***	***p***
ACB_bombus ~ pair + RwP + PC1_size + PC2_size + (1∣sp)	pair	9	727.57	2.38	0.012
ACB_bombus ~ pair + RwP + PC1_size + PC2_size + (1∣sp)	RwP	2	85.33	1.57	0.215
ACB_bombus ~ pair + RwP + PC1_size + PC2_size + (1∣sp)	PC1_size	1	90.06	2.04	0.157
ACB_bombus ~ pair + RwP + PC1_size + PC2_size + (1∣sp)	PC2_size	1	88.07	13.85	0.000
ACB_bombus ~ pair + RwT + PC1_size + PC2_size + (1∣sp)	pair	9	726.81	2.36	0.012
ACB_bombus ~ pair + RwT + PC1_size + PC2_size + (1∣sp)	RwT	4	82.17	1.28	0.284
ACB_bombus ~ pair + RwT + PC1_size + PC2_size + (1∣sp)	PC1_size	1	89.68	1.64	0.204
ACB_bombus ~ pair + RwT + PC1_size + PC2_size + (1∣sp)	PC2_size	1	83.16	9.01	0.004
CCB_bombus ~ pair + RwP + PC1_size + PC2_size + (1∣sp)	pair	9	726.58	18.71	0.000
CCB_bombus ~ pair + RwP + PC1_size + PC2_size + (1∣sp)	RwP	2	88.16	1.47	0.236
CCB_bombus ~ pair + RwP + PC1_size + PC2_size + (1∣sp)	PC1_size	1	93.17	12.94	0.001
CCB_bombus ~ pair + RwP + PC1_size + PC2_size + (1∣sp)	PC2_size	1	90.45	2.03	0.157
CCB_bombus ~ pair + RwT + PC1_size + PC2_size + (1∣sp)	pair	9	726.34	18.69	0.000
CCB_bombus ~ pair + RwT + PC1_size + PC2_size + (1∣sp)	RwT	4	86.01	0.93	0.451
CCB_bombus ~ pair + RwT + PC1_size + PC2_size + (1∣sp)	PC1_size	1	93.92	12.70	0.001
CCB_bombus ~ pair + RwT + PC1_size + PC2_size + (1∣sp)	PC2_size	1	86.49	2.24	0.138
ACB_bombus ~ pair + RwP + PC1_size + PC2_size + (1∣sp)	pair	9	727.57	2.38	0.012
ACB_bombus ~ pair + RwP + PC1_size + PC2_size + (1∣sp)	RwP	2	85.33	1.57	0.215

ACB_bombus ~ pair + RwP + PC1_size + PC2_size + (1|sp)	PC1_size	1	90.06	2.04	0.157
ACB_bombus ~ pair + RwP + PC1_size + PC2_size + (1|sp)	PC2_size	1	88.07	13.85	0.000
ACB_bombus ~ pair + RwT + PC1_size + PC2_size + (1|sp)	pair	9	726.81	2.36	0.012
ACB_bombus ~ pair + RwT + PC1_size + PC2_size + (1|sp)	RwT	4	82.17	1.28	0.284
ACB_bombus ~ pair + RwT + PC1_size + PC2_size + (1|sp)	PC1_size	1	89.68	1.64	0.204
ACB_bombus ~ pair + RwT + PC1_size + PC2_size + (1|sp)	PC2_size	1	83.16	9.01	0.004
CCB_bombus ~ pair + RwP + PC1_size + PC2_size + (1|sp)	pair	9	726.58	18.71	0.000
CCB_bombus ~ pair + RwP + PC1_size + PC2_size + (1|sp)	RwP	2	88.16	1.47	0.236
CCB_bombus ~ pair + RwP + PC1_size + PC2_size + (1|sp)	PC1_size	1	93.17	12.94	0.001

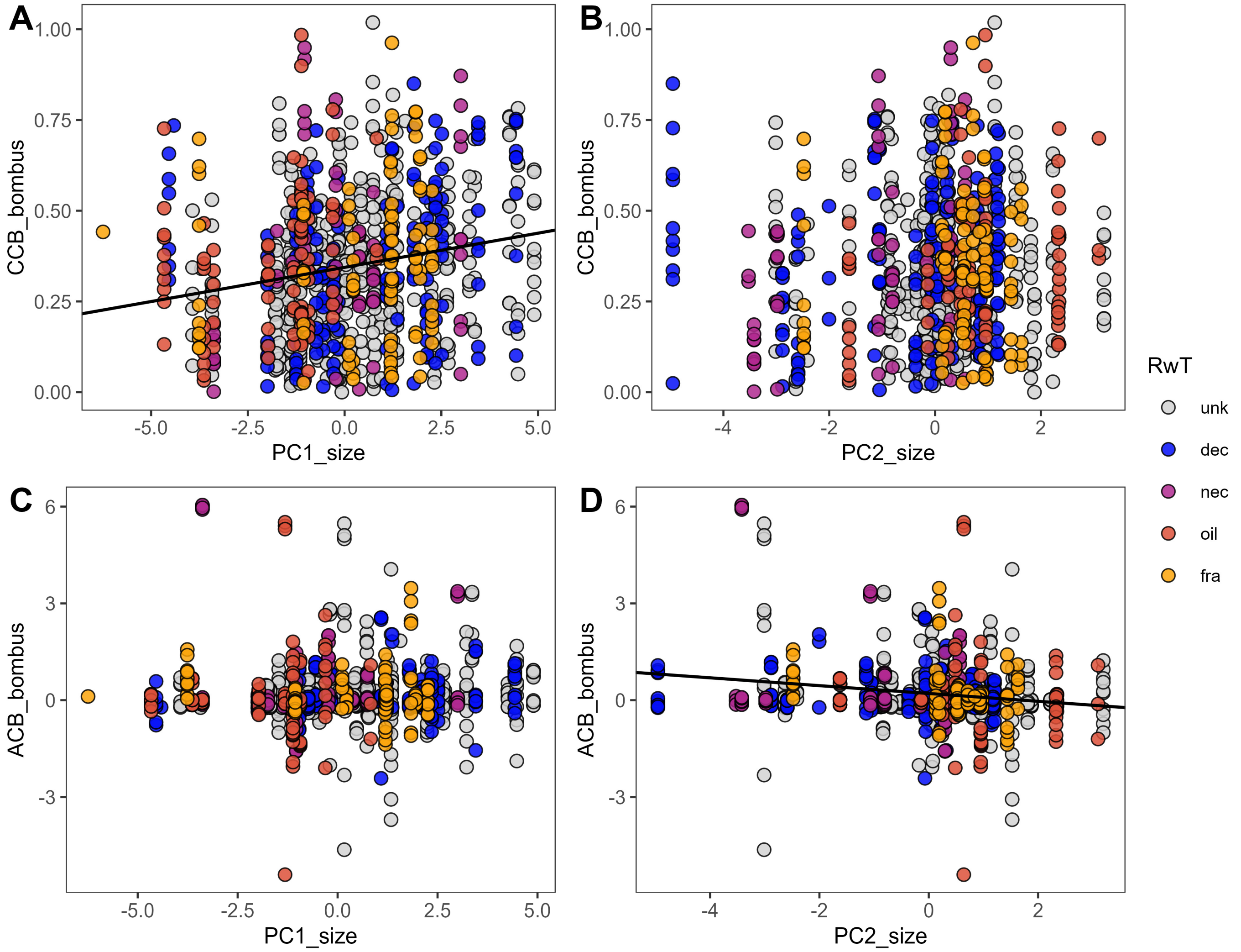

Figure S5: Linear mixed-effect models showing the associations between flower (sepal, petal, lip tips and lip bases) chromatic (CCB) and achromatic contrasts (ACB) in bee vision and flower and display size.

Table S6. Pollinator body size dataset for bee-pollinated orchids. For each orchid species, we list the bee families, tribes, and identified bee visitors compiled from Ackerman et al. 2023 [1] and references therein. Pollinator size is summarized as the mean intertegular span (ITS; mm), compiled from the literature (Table SM7) across all recorded visitor species for each orchid (i.e., species-level average of bee body size), and is used as a proxy for pollinator size in subsequent analyses.

orchid species	bee families	bee tribes	bee species	mean ITS (mm)
Arundina graminifolia	Apidae, Halictidae, Megachilidae, Scoliidae, Vespidae	Apini, Campsomerini, Centridini, Eumenini, Halictini, Megachilini, Melectini	*Apis dorsata, Apis mellifera, Centris haemorrhoidalis, Megacampsomeris mojiensis, Megachile yaeyamaensis, Nomia pavonura, Rhynchium quinquecinctum, Thyreus tadaonis, Xylocopa* sp.	3,86
Catasetum cernuum	Apidae	Euglossini	*Eufriesea violacea*	4,45
Catasetum fimbriatum	Apidae	Euglossini	*Efriesea combinata, Efriesea violacea, Efriesea violacens, Eufriesea auriceps, Eulaema cingulata*	5,47
Catasetum luridum	Apidae	Euglossini	*Euglossa cordata*	2,66
Catasetum pileatum	Apidae	Euglossini	*Eulaema bombiformis, Eulaema cingulata, Eulaema meriana, Eulaema nigrita, Eulaema peruviana, Eulaema seabrai*	6,09
Coelogyne flaccida	Apidae	Apini	*Apis cerana*	2,67
Coelogyne viscosa	Apidae	Apini	*Apis cerana*	2,67
Cycnoches haagii	Apidae	Euglossini	*Euglossa* sp.	3,15
Cycnoches pentadactylon	Apidae	Euglossini	*Eufriesea superba*	5,07
Cymbidium aloifolium	Apidae	Apini	*Apis cerana*	2,67
Cyrtopodium flavum	Apidae	Centridini	*Centris nitida*	4,23
Dendrobium anosmum	Apidae	Apini	*Apis cerana, Apis dorsata*	3,29
Dendrobium kingianum	Apidae, Halictidae	Apini, Augochlorini, Meliponini	*Apis mellifera, Trigona* sp.	2,72
Dendrobium loddigesii	Apidae	Ctenoplectrini	*Ctenoplectra florisomnis*	3,00
Dendrobium nobile	Apidae	Ctenoplectrini	*Ctenoplectra cornuta*	3,00
Dendrobium speciosum	Apidae, Colletidae, Halictidae	Apini, Euryglossini, Halictini, Hylaeini, Meliponini	*Apis mellifera, Euryglossa* sp., *Hylaeus* sp., *Lasioglossum brisbanensis, Trigona carbonaria*	2,00
Dichaea pendula	Apidae	Euglossini	*Eufriesea violacea, Euglossa* sp.	3,80
Encyclia cordigera	Apidae	Xylocopini	*Xylocopa nautlana, Xylocopa* sp.	6,23
Encyclia mapuerae	Apidae, Bembicidae, Vespidae	Centridini, Epiponini, Gorytini	*Centris varia, Rubrica nasuta, Stelopolybia pallipes*	5,13
Gomesa flexuosa	Apidae	Centridini	*Centris trigonoides*	3,49

Gongora bufonia	Apidae	Euglossini	*Eufriesea violacea*	4,45
Ionopsis utricularioides	Apidae, Halictidae	Augochlorini, Ceratinini, Halictidae, Meliponini, Tapinotaspidini	*Augochlora* sp., *Ceratina* sp., *Nannotrigona testaceicornis, Paratetrapedia flaviola, Paratrigona lineata*	3,03
Lockhartia lunifera	Apidae	Tetrapediini	*Tetrapedia* sp.	2,23
Maxillaria chrysantha	Apidae	Meliponini	*Trigona* sp.	2,17
Myrmecophila tibicinis	Apidae	Euglossini, Xylocopini	*Eulaema polychroma, Xylocopa* sp.	6,11
Oncidium ornithorhyncum	Apidae	Centridini	*Centris* sp.	5,13
Papilionanthe teres	Apidae	Xylocopini	*Xylocopa latipes*	6,23
Polystachya pubescens	Apidae, Halictidae	Halictini, Meliponini, Tapinotaspidini	*Dialictus creberrimus, Lasioglossum emirnense, Lasioglossum nitididorsatum, Melipona* sp., *Paratetrapedia fervida, Plebeya droryana, Tetragonisca angustula, Trigona spinipes*	2,09
Pseudolaelia corcovadensis	Apidae	Bombini	*Bombus atratus*	4,14
Spathoglottis unguiculata	Apidae	Xylocopini	*Xylocopa* sp.	6,23
Tolumnia variegata	Apidae	Centridini	*Centris decolorata*	5,13
Trichocentrum jonesianum	Apidae	Centridini	*Epicharis* sp.	5,10
Trichocentrum lanceanum	Apidae	Centridini	*Centris* sp.	5,13
Vanda tricolor	Apidae	Xylocopini	*Xylocopa latipes*	6,23
Warrea warreana	Apidae	Bombini	*Bombus brasiliensis*	4,38
Zygopetalum maculatum	Apidae	Bombini, Centridini, Xylocopini	*Bombus* sp., *Centris confusa, Xylocopa* sp.	5,17

Table S7: Intertegular span data in mm for 744 bee species compiled from [2–8]. Genus followed by "sp" are genus-level means.

References

1. Ackerman, J.D.; Phillips, R.D.; Tremblay, R.L.; Karremans, A.; Reiter, N.; Peter, C.I.; Bogarín, D.; Pérez-Escobar, O.A.; Liu, H. Beyond the Various Contrivances by Which Orchids Are Pollinated: Global Patterns in Orchid Pollination Biology. *Bot. J. Linn. Soc.* **2023**, *202*, 295–324, doi:10.1093/botlinnean/boac082.
2. Bullock, S.H. Relationships among Body Size, Wing Size and Mass in Bees from a Tropical Dry Forest in México. *J. Kans. Entomol. Soc.* **1999**, *72*, 426–439.
3. Burkart, A.; Lunau, K.; Schlindwein, C. Comparative Bioacoustical Studies on Flight and Buzzing of Neotropical Bees. *J. Pollinat. Ecol.* **2012**, *6*, doi:10.26786/1920-7603(2011)17.
4. Kendall, L.K.; Rader, R.; Gagic, V.; Cariveau, D.P.; Albrecht, M.; Baldock, K.C.R.; Freitas, B.M.; Hall, M.; Holzschuh, A.; Molina, F.P.; et al. Pollinator Size and Its Consequences: Robust Estimates of Body Size in Pollinating Insects. *Ecol. Evol.* **2019**, *9*, 1702–1714, doi:10.1002/ece3.4835.

5. Gomes, I.; Silva, V.; Gonçalves, R.; Ordóñez-Parra, C.; Procópio-Santos, C.; Queroz, S.; Castro, D.; Pena, J.; Maruyama, P. Exploring the Determinants of Bee Diversity in Tropical Urban Areas and Their Implications for Conservation. *Landsc. Urban Plan.* **2025**, *263*, 105440, doi:10.1016/j.landurbplan.2025.105440.
6. Santos, C.; Serrão, J. Histology Of The Ileum In Bees (Hymenoptera, Apoidea). *Braz. J. Morphol. Sci.* **2006**, *23*, 405–413.
7. Ferreira, V.G.S.; Aguiar, C.M.L.; Rebouças, P.L. de O.; Martins, H.O. de J.; Francoy, T.M. Influence of trap nest dimensions on reproductive biology and offspringbody size in two centris bee species (hymenoptera: apidae). *J. Apic. Res.* **2025**, *No 2025*, 01–11.
8. Becco, M.G. Padrões de variação de tamanho corporal entre espécies de abelhas nativas do Estado do Ceará: distância intertegular como medida comparativa. **2021**.
